# Prioritizing conserved areas threatened by wildfire and fragmentation for monitoring and management

**DOI:** 10.1371/journal.pone.0200203

**Published:** 2018-09-07

**Authors:** Jeff A. Tracey, Carlton J. Rochester, Stacie A. Hathaway, Kristine L. Preston, Alexandra D. Syphard, Amy G. Vandergast, Jay E. Diffendorfer, Janet Franklin, Jason B. MacKenzie, Tomas A. Oberbauer, Scott Tremor, Clark S. Winchell, Robert N. Fisher

**Affiliations:** 1 U.S. Geological Survey, Western Ecological Research Center, San Diego, California, United States of America; 2 San Diego Management and Monitoring Program, San Diego, California, United States of America; 3 Conservation Biology Institute, La Mesa, CA, United States of America; 4 Geosciences and Environmental Change Science Center, U.S. Geological Survey, Denver, Colorado, United States of America; 5 Department of Botany and Plant Sciences, University of California, Riverside, California, United States of America; 6 Environment and Planning Directorate, ACT Government, Canberra, Australia; 7 AECOM, San Diego, California, United States of America; 8 San Diego Natural History Museum, San Diego, California, United States of America; 9 U.S. Fish & Wildlife Service, Carlsbad, California, United States of America; Chinese Academy of Forestry, CHINA

## Abstract

In many parts of the world, the combined effects of habitat fragmentation and altered disturbance regimes pose a significant threat to biodiversity. This is particularly true in Mediterranean-type ecosystems (MTEs), which tend to be fire-prone, species rich, and heavily impacted by human land use. Given the spatial complexity of overlapping threats and species’ vulnerability along with limited conservation budgets, methods are needed for prioritizing areas for monitoring and management in these regions. We developed a multi-criteria Pareto ranking methodology for prioritizing spatial units for conservation and applied it to fire threat, habitat fragmentation threat, species richness, and genetic biodiversity criteria in San Diego County, California, USA. We summarized the criteria and Pareto ranking results (from west to east) within the maritime, coastal, transitional, inland climate zones within San Diego County. Fire threat increased from the maritime zone eastward to the transitional zone, then decreased in the mountainous inland climate zone. Number of fires and fire return interval departure were strongly negatively correlated. Fragmentation threats, particularly road density and development density, were highest in the maritime climate zone, declined towards the east, and were positively correlated. Species richness criteria showed distributions among climate zones similar to those of the fire threat variables. When using species richness and fire threat criteria, most lower-ranked (higher conservation priority) units occurred in the coastal and transitional zones. When considering genetic biodiversity, lower-ranked units occurred more often in the mountainous inland zone. With Pareto ranking, there is no need to select criteria weights as part of the decision-making process. However, negative correlations and larger numbers of criteria can result in more units assigned to the same rank. Pareto ranking is broadly applicable and can be used as a standalone decision analysis method or in conjunction with other methods.

## Introduction

Considerable effort has been put into identifying land that could be placed in conservation reserves and acquiring such lands. However, even once land has been conserved, numerous threats may impact biodiversity within reserves. Threats include invasive species, infrastructure, pollution, recreation, and disturbances. Conservation practitioners and land managers typically have limited budgets and other resources that prevent them from monitoring and managing all threats in all places. Focusing limited resources for monitoring and management when or where they will be most productive is ideal. This can be accomplished if areas are prioritized, particularly within conserved lands, for monitoring and management or for research on the impacts of disturbance on biodiversity. The need to prioritize areas is a common problem in conservation.

In this paper, we present a methodology for prioritizing areas where monitoring and management efforts could be most productive using available spatial attributes related to biodiversity and threats. Our approach consists of four steps. First, we define the spatial units to be prioritized. We represent space as a raster of grid cells that serve as alternatives in the prioritization process. Second, we develop attributes describing species and genetic biodiversity and threats within each grid cell. Because we have more than one attribute for each cell, this is a multi-criteria decision analysis (MCDA) problem [1, 2]. We use species distribution models (SDMs) to estimate the occurrence of target species and to represent an index of biodiversity. We further reduce SDMs for many species into attributes for a smaller number of taxonomic groups by summing the SDMs for species to estimate the biodiversity index within each taxon. Third, we apply *value functions* (or *utility functions*) to the grid cell attributes to produce criteria that will be used for prioritization. Finally, we prioritize grid cells using all of the criteria with Pareto ranking.

Numerous MCDA methods are available (see [3–5]), and they have been classified into *single synthesizing criterion*, *outranking*, and *mixed* methods [3]. In single synthesizing criterion methods, criteria are aggregated into a single criterion before ranking the alternatives [3]. The use of single synthesizing criterion methods (SCMs) has overtaken the use of outranking methods [5]. Within SCMs, multi-attribute value theory (MAVT) and analytic hierarchical process (AHP) are the most commonly used [5]. Indeed, they have been applied in conservation and natural resource management [1, 4, 6–9]. Both methods require some consensus on subjectively selected weights (MVAT, [5, 9]) or relative importance values (AHP, [10, 11]). Discussion about the relative importance of different criteria can help build a common understanding of the decision [1, 12]; however, it can also be a lengthy, subjective, and contentious process.

Pareto ranking provides an alternative method that does not rely on subjective weights applied to the criteria in the decision analysis algorithm and does not aggregate multiple criteria into a single value before ranking alternatives. When ranking on multiple criteria, alternatives can be compared using the concept of Pareto dominance. Suppose, for example, that we are comparing multiple criteria for two alternatives. The first alternative may be at least as good with respect to all criterion as the second and strictly better in at least one criterion. In this case, we say that the first alternative *dominates* the second [13]. However, it is also possible that the first alternative is better than the second for some criteria, but the second alternative is better than the first for some of the others. In this case, each alternative is considered *non-dominated*. For a non-dominated alternative, no other alternative is at least as good or better with respect to all criteria. Selecting one non-dominated alternative over another involves a trade-off in one or more criteria (they are *Pareto optimal*). In a MCDA, non-dominated alternatives should always be selected over dominated ones [4]. If we extend this idea to the comparison of a larger set of alternatives, we can identify those alternatives that are non-dominated after comparison to all others, which is called the *non-dominated set* (NDS). We can assign each alternative in this first NDS a rank of 1. If we find the NDS among the alternatives that have not been assigned a rank, we can assign the elements of this NDS a rank of 2. This can be repeated, increasing the rank each time and ignoring the alternatives that have already been ranked, until all of the alternatives have been ranked. This process is known as Pareto ranking [8, 13].

Pareto ranking has several characteristics relative to other MCDA approaches. First, as previously mentioned, there is no need for subjectively-selected weights on criteria. Second, since criteria are not aggregated into a single criterion prior to ranking, they do not need to be converted to a common scale. This can be helpful if the MCDA involves very different criteria that are not comparable. Third, the Pareto ranking algorithm is conceptually simple. Pareto ranking can assign the same rank to many alternatives; therefore, depending on the problem, additional prioritization or stakeholder discussion may be required. However, these finer-resolution decisions will be narrowed down to within non-dominated sets of alternatives. For problems such as the one presented in this paper, where we are selecting some proportion of a large set of alternatives, additional prioritization may be unnecessary. Pareto ranking has been used in a wide range of fields, particularly in evolutionary computing to find solutions to complex problems [13]. Although Pareto ranking has had some use in conservation and natural resource management [8, 14], it has unrealized potential to prioritize spatial units for conservation, monitoring, and management.

**Fig 1:**
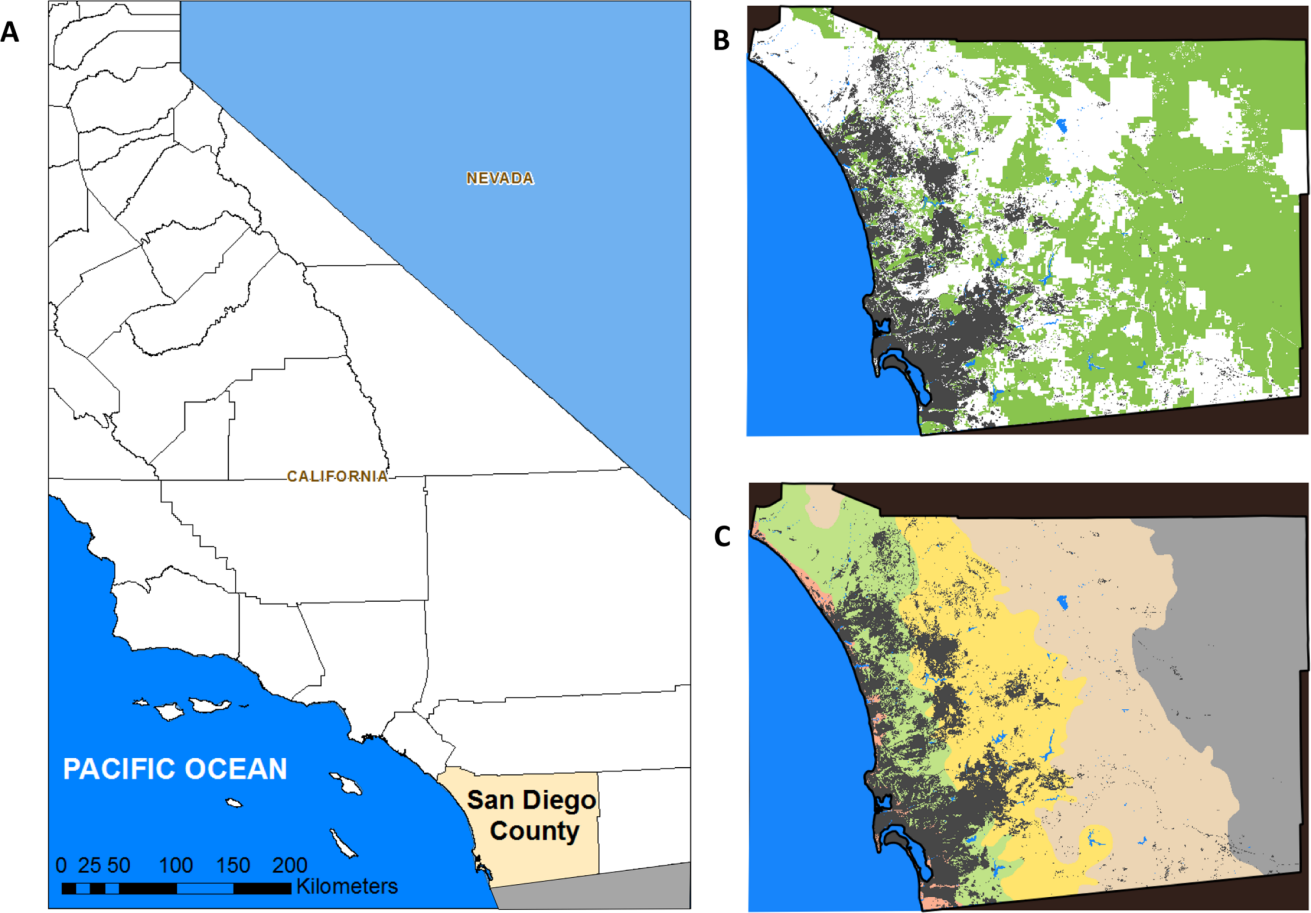
Study area, climate zones, and conserved lands. Our study area is San Diego County, USA (A). Under California’s Natural Communities Conservation Planning program, efforts have been made to build a network of conserved lands (B). Conserved lands are shown in green and non-conserved areas are shown in white. The region has been divided into five climate zones (C): maritime (light red), coastal (light green), transitional (yellow), inland (tan), and desert (gray). In (B) and (C), urban areas are shown in dark gray and water is shown in dark blue.

Our objective is to demonstrate this approach for prioritizing spatial units by applying it to criteria related to biodiversity and threats from wildfire and habitat fragmentation to address conservation planning needs in San Diego County, California, USA (Fig 1A). This region has high biodiversity that has been eroded by both habitat fragmentation and anthropogenic changes in fire regimes. According to The Nature Conservancy, San Diego County is the most biologically rich county in the continental United States [15]. Due to a large and growing human population, natural habitats in many parts of the region have been severely fragmented by urban and exurban development, roads, infrastructure, and agriculture (Fig 1B). As a result, San Diego County has become a hotspot for threatened and endangered species in the United States [16]. Most of the natural lands in southern California are currently experiencing frequent fires that exceed the resilience of many native species [16]. The ignition of most of these fires is attributed to human actions [17]. In 2003 and 2007, San Diego County experienced two of the state’s largest historical wildfires, with total areas of 155,600 and 149,930 ha, respectively. Large wildfires have also increased in frequency as the growing human population encroaches into the wildlands [18, 19]. This increasing fire frequency may negatively affect both plant [20, 21] and animal biodiversity [22, 23]. In order to conserve the unique biodiversity and ecological integrity of San Diego County, extensive effort has been invested in the development of Habitat Conservation Plans (HCPs) under the Federal Endangered Species Act and the State’s Natural Community Conservation Planning (NCCP) Acts of 1991 and 2003 [24]. In order to efficiently use limited resources, regional conservation planners needed to prioritize the areas according to their biodiversity and threats. We applied our Pareto ranking-based approach to support regional planning for monitoring and management.

## Methods

### Study area

San Diego County, California has an area of approximately 10,912 km^2^ (Fig 1). Based on distance from the coast, elevation, topography, and climate variables, the county has been classified into maritime, coastal, transitional, inland, and desert climate zones [25] (Fig 1C). We used these climate zones to aid in our interpretation of the results. The County had, as of 2008, a population of more than 3.1 million people and a projected population of 4.4 million by 2050 according to the San Diego Association of Governments (SANDAG). This large and growing human population has resulted in 14 percent of the County’s approximately 10,912 km^2^ being classified as urban. Of the County’s non-urbanized land, approximately 46 percent has been placed in some form of conserved status (Fig 1B).

Our analysis was performed over the extent of San Diego County, excluding urban, water, and desert areas. We constructed a spatial analysis mask by excluding all areas outside of San Diego County, all urban areas, and all bodies of water based on the wildlife habitat relations type (WHRTYPE) in the CalVeg vegetation cover data [26], and the desert climate zone based on the SANDAG climate zone classification. The desert was omitted because data collection for species occurrence was biased toward non-desert areas and fires occur less frequently in the desert climate zone. The polygon data for the analysis mask was rasterized at 50-m resolution (i.e. 0.25 ha cells), a resolution that we determined provided a balance between an acceptable representation of the size and shape of smaller habitat patches in our landscape and minimizing the number of spatial units (i.e. cells) to be ranked. Attributes used for prioritization were developed for each of these grid cells.

### Spatial attributes for prioritization

We developed spatial attributes related to indicators of high biodiversity and threats to that biodiversity. Attributes for threats to biodiversity included three wildfire-related metrics and three habitat fragmentation-related metrics (Table 1 and S1 Appendix). The three fire-related attributes were the number of historical fires, which indicates overall fire frequency over the last century; mean predicted probability of the ignition of large fires, which indicates the potential for new fires being started; and, mean fire return interval departure, which indicates the change of estimated fire inter-event times (years between fires) for the historical time period compared to the pre-settlement times [27]. The three fragmentation-related attributes were development density, the density of all roads, and fragment area. For species diversity, we used predictions from species distribution models (SDMs; Table 2 and S1 Appendix). We used SDMs that were developed for other applications and had some level of model evaluation and/or peer review. We combined the SDMs within plant, herpetofauna (amphibian and reptile), bird, and mammal taxonomic groups following an additive model, that is, we summed the habitat suitability indices estimated by SDMs (on a scale of 0–1) for each animal species within each taxon. (For plants the SDMs were thresholded to produce binary maps of predicted occurrence and so the sum represents relative species richness based on ~9% of the regional flora.) This is straightforward because all of the SDMs are, unlike many of the other attributes we use, in the same “currency” and we assumed that all species within a taxonomic group are equally important and should be given equal weight. More importantly, this yielded an index of relative biodiversity, for each taxonomic group. If desired, however, maps of habitat suitability for species within taxonomic groups could be weighted based on their perceived importance before summing. From the SDMs we produced four attributes: the biodiversity index for the taxa modeled within the plant, herpetofauna, bird, and mammal taxonomic groups. As a compliment to species richness, we also used predicted genetic diversity and genetic divergence calculated for 21 small invertebrate and vertebrate animal species to represent genetic biodiversity [28] (Table 2 and S1 Appendix). The genetic variation within species provides the raw material underlying a species’ ability to adapt to future environmental change. Localized areas of high genetic diversity and divergence may be indicative of large and stable populations, (providing evolutionary resilience) [29–31], or of recontact zones between previously isolated lineages [32, 33]. Preservation of these regions and movement corridors to and from them may be important for allowing for traits and diversity to spread through a species range. The most comprehensive approach to conservation prioritization would be to prioritize both regions with high species richness and regions with high genetic diversity.

**Table 1:** Threat-related attributes used to rank the grid cells. For each variable, the subscript *k* indexes a grid cell that is being ranked against the other cells. These data are available in raster format at https://doi.org/10.5066/P95LH274.

Description
*Number of Historical Fires (F*_*k*,*Nfires*_*)—*We rasterized fire perimeters from the statewide CalFire database of wildfires ≥ 10 acres for 1910–2012 at a 50 meter resolution, then added the fire perimeter rasters to compute the total fire number of fires per cell from 1910 to 2012. Reference: Fire perimeters [34]
*Probability of Ignition (F*_*k*,*pr(ign)*_*)—*We used generalized additive model (GAM) predictions for the probability of ignition of large fires that were made using point data for ignition locations. Reference: [17]
*Fire Return Interval Departure (F*_*k*,*FRID*_*)—*We used USFS estimates of fire return interval departure (FRID). We used MedianFreqDep, which is a measure of the departure of the current fire return interval from the median reference fire return interval. A negative value for MedianFrepDep indicates that intervals between recorded fires in the last century are shorter than they were estimated to be in the pre-Euroamerican fire regime. References: [27]
*Road Density (H*_*k*,*road*_*)—*Using the SANDAG ‘all road' line data rasterized at a 50 m resolution, we calculated the proportion of grid cells within a 500 meter radius of the center of each grid cell that were intersected by roads in the all roads layer. References: SANGIS all roads shapefile [25]
*Development Density (H*_*k*,*dev*_*)—*We calculated development density using the CalVeg land cover data for urban areas and agricultural areas that included cropland, orchards, eucalyptus groves, and pasture. For each grid cell, we described the impact of urban and agricultural development as [(number of urban cells within 500 m) + 0.3*(number of agriculture cells within 500m)]/(total number of cells within 500 m). References: CalVeg [26]
*Fragment Area (H*_*k*,*patch*_*)—*Patch area (in hectares) was calculated as an attribute for patches of continuous road-free, non-urban areas. In this case, SANDAG ‘all roads’ data were used to delineate road-free patches. The fragment area of the patch was assigned to each grid cell in the fragment. References: CalVeg [26], SANGIS all roads shapefile [25]

**Table 2:** Biodiversity-related attributes used to rank the cells. For each variable, the subscript *k* indexes a grid cell that is being ranked against the other cells. These data are available in raster format at https://doi.org/10.5066/P95LH274.

Description
*Index of Plant Biodiversity (S*_*k*,*plant*_*)—*We used SDMs for 138 plant species that were developed by The Nature Conservancy using a maximum entropy (MaxEnt) approach [35, 36]. The continuous probabilities of presence were converted to binary scores (1 = suitable, 0 = unsuitable) using thresholds that maximized the recovery of both true positives and true negatives for each species in testing data [37]. The binary scores for the 138 plant species were summed to compute predicted plant species richness. Reference: [36]
*Index of Reptile and Amphibian (herpetofauna) Biodiversity (S*_*k*,*herp*_*)—*We used SDMs (random forest or generalized additive models) developed by Franklin et al. [38] for 24 reptiles and 5 amphibians. The probability of presence for the 29 species was summed to yield predicted herpetofauna species richness. Reference: [38]
*Index of Bird Biodiversity (S*_*k*,*bird*_*)—*We used SDMs (partitioned Mahalanobis D2 models) developed by KLP for 6 bird species. Habitat similarity index scores from 0 (low suitability) to 1 (high suitability) were summed to yield predicted bird species richness. Reference: Unpublished
*Index of Mammal Biodiversity (S*_*k*,*mamm*_*)—*We used SDMs for 18 mammal species ranging from small mammals to mountain lions developed by JED and ST using ecological niche factor analysis, implemented in Biomapper [39]. The resulting suitability scores were rescaled on 0.0 to 1.0 and summed to yield predicted mammal richness. Reference: [40]
*Mean Genetic Diversity (G*_*k*,*divers*_*)—*We used previously developed raster models of intra-population sequence diversity averaged across 14 small animal species (invertebrates, amphibians, reptiles, birds and mammals). We interpolated continuous rasters from average sequence diversity measured within populations for the 14 species (S1 Appendix) for which mtDNA sequence data were collected throughout southern California. These were averaged into a single raster depicting regional hotspots of intrapopulation genetic diversity for the species assemblage. Reference: [28]
*Mean Genetic Divergence (G*_*k*,*diverg*_*)—*A similar approach was used to create a raster depicting regional hotspots of average genetic divergence for 21 small animal species (invertebrates, amphibians, reptiles, birds and mammals). Sequence divergence between pairs of populations was mapped to the geographic midpoints between populations and interpolated into continuous rasters the 21 species (S1 Appendix) and averaged into a single layer. Reference: [28]

### Cell-level criteria

First, we will provide some general notation for the criteria and then describe the setup for our specific problem. We prioritize each grid cell based on a vector of attributes associated with it. We index the grid cell rows by *i* = 1, …, *I* and the columns by *j* = 1, …, *J*. Thus, we can re-index the grid cells by *k* = (*i*– 1)×*J* + *j*, where k = 1, …, *K* = *I×J*. Furthermore, we index the attributes or the criteria based on them by *m* = 1, …, *M*, where *M* is the number of criteria considered in the ranking. The vector of attributes for the *k*^*th*^ grid cell is denoted as **a**_*k*_ = (*a*_*k*,1_,…,*a*_*k*,*M*_). The vector of all values for the *m*^*th*^ attribute across all grid cells is denoted by **a**_*m*_ = (*a*_1,*m*_,…,*a*_*K*,*m*_)^*T*^.

Attributes are typically transformed into criteria reflecting our preferences by a *value function* prior to ranking. Formulating these functions is easy in Pareto ranking, because attributes do not need to be converted to a common scale and only the comparative values (<, = , or >) of the criteria produced by the value functions matter. These value functions may, for example, change the sign of the variable so that a smaller value indicates greater preference and/or transform the variable so that it is non-negative. Generally, this transformation can be denoted as *x*_*k*,*m*_ = *f*_*m*_(*a*_*k*,*m*_), which produces the vector of criteria **x**_*k*_ = (*x*_*k*,1_,⋯,*x*_*k*,*M*_) for grid cell *k* and the column-vector of values for the *m*^*th*^ criterion across all grid cells **x**_*m*_ = (*x*_1,*m*_,⋯,*x*_*K*,*m*_)^*T*^. Because we express Pareto ranking below as a minimization problem, we transformed the attributes (using the value functions) such that lower values of a criterion indicate a higher preference (i.e. a grid cell that requires greater management attention).

In our specific application, for the *k*^*th*^ grid cell we have three attributes related to fire threats (*F*_*k*,*Nfires*_, *F*_*k*,*pr(ign)*_, and *F*_*k*,*FRID*_), three attributes related to habitat fragmentation (*H*_*k*,*road*_, *H*_*k*,*dev*_, and *H*_*k*,*patch*_), four attributes related to species diversity (S_k,plant_, S_k,herp_, S_k,bird_, and S_k,mamm_), and two attributes related to genetic biodiversity (*G*_*k*,*divers*_ and *G*_*k*,*diverg*_). We denote the vectors of these attributes as **F**_*k*_, **H**_*k*_, **S**_*k*_, and **G**_*k*_, respectively. The column-vector of a given attribute for all grid cells is denoted by **F**_*Nfires*_ = (*F*_*1*, *Nfires*_, …, *F*_*I×J*,*Nfires*_)^T^, **F**_pr(ign)_ = (*F*_*1*, *pr(ign)*_, …, *F*_*I×J*, *pr(ign)*_)^T^, and so on for all other criteria (Tables 3 and 4). The column-vectors of related attributes are then combined to form a matrix; for example, the fire-related attributes are combined as **F** = [**F**_*Nfires*_ | **F**_*pr(ign)*_ | **F**_*FRID*_] (see Tables 3 and 4) where “[**A** | **B**]” denotes forming an augmented matrix by column-wise concatenation of vectors or matrices **A** and **B**. Prior to ranking, we applied either the value function

$$x_{k,m}=f(a_{k,m})=[a_{k,m}-\min(a_{m})]/[\max(a_{m})-\min(a_{m})]$$

or the value function

$$x_{k,m}=g(a_{k,m})=[\max(a_{m})-a_{k,m}]/[\max(a_{m})-\min(a_{m})]$$

to each column-vector of attributes to ensure that (1) an attribute with a higher preference was transformed into criterion with a smaller value and (2) the values of all criteria were non-negative (see Tables 3 and 4). Specifically, we denote the matrices of related criteria as **F**′ = [*g*(**F**_*Nfires*_) | *g*(**F**_*pr(ign)*_) | *f*(**F**_*FRID*_**)**], **H**′ = [*g*(**H**_*road*_) | *g*(**H**_*dev*_) | *f*(**H**_*patch*_)], **S**′ = [*g*(**S**_*plant*_) | *g*(**S**_*herp*_) | *g*(**S**_*bird*_) | *g*(**S**_*mamm*_)], and **G**′ = [*g*(**G**_*divers*_) | *g*(**G**_*diverg*_)]. Using these four matrices of related criteria, we formed six alternative decision matrices of criteria for the grid cells so that we could explore the effects of using different criteria in the ranking process: (1) fire and species criteria matrix denoted [**F**′ | **S**′], (2) habitat fragmentation and species criteria matrix [**H**′ | **S**′], (3) fire and genetic criteria matrix [**F**′ | **G**′], (4) habitat fragmentation and genetic criteria matrix [**H**′ | **G**′], (5) fire, species, and genetic criteria matrix [**F**′ | **S**′ | **G**′], and (6) habitat fragmentation, species, and genetic criteria matrix [**H**′ | **S**′ | **G**′]. In these matrices, each column corresponds to a single criterion and each row corresponds to a grid cell. If the values of one or more criteria were missing for a grid cell in a given criteria matrix, that cell was not ranked.

**Table 3:** Threat criteria organized into matrices. Each column corresponds to a single criterion across all grid cells and each row corresponds to multiple criteria for a single grid cell. Related criteria are grouped into matrices: **F** and **H** are matrices of fire and habita criteria, respectively.

	F			H		
Cell (*k*)	F_*Nfires*_	F_*pr(ign)*_	F_*FRID*_	H_*road*_	H_*dev*_	H_*patch*_
1	*F*_*1*,*Nfires*_	*F*_*1*,*pr(ign)*_	*F*_*1*,*FRID*_	*H*_*1*,*road*_	*H*_*1*,*dev*_	*H*_*1*,*patch*_
2	*F*_*2*,*Nfires*_	*F*_*2*,*pr(ign)*_	*F*_*2*,*FRID*_	*H*_*2*,*road*_	*H*_*2*,*dev*_	*H*_*2*,*patch*_
…	*…*	*…*	*…*	*…*	*…*	*…*
*I*×*J*	*F*_*I×J*,*Nfires*_	*F*_*I×J*,*pr(ign)*_	*F*_*I×J*,*FRID*_	*H*_*I×J*,*road*_	*H*_*I×J*,*dev*_	*H*_*I×J*,*patch*_

**Table 4:** Biodiversity criteria organized into matrices. Each column corresponds to a single criterion across all grid cells and each row corresponds to multiple criteria for a single grid cell. Related criteria are grouped into matrices: **S** and **G** are matrices of species and genetic criteria, respectively.

	S				G	
Cell (*k*)	S_*plant*_	S_*herp*_	S_*bird*_	S_*mamm*_	G_*diverse*_	G_*diverge*_
1	*S*_*1*,*plant*_	*S*_*1*,*herp*_	*S*_*1*,*bird*_	*S*_*1*,*mamm*_	*G*_*1*,*divers*_	*G*_*1*,*diverg*_
2	*S*_*2*,*plant*_	*S*_*2*,*herp*_	*S*_*2*,*bird*_	*S*_*2*,*mamm*_	*G*_*2*,*divers*_	*G*_*2*,*diverg*_
…	*…*	*…*	*…*	*…*	*…*	*…*
*I*×*J*	*S*_*I×J*,*plant*_	*S*_*I×J*,*herp*_	*S*_*I×J*,*bird*_	*S*_*I×J*,*mamm*_	*G*_*I×J*,*divers*_	*G*_*I×J*,*diverg*_

### Pareto ranking

**Fig 2:**
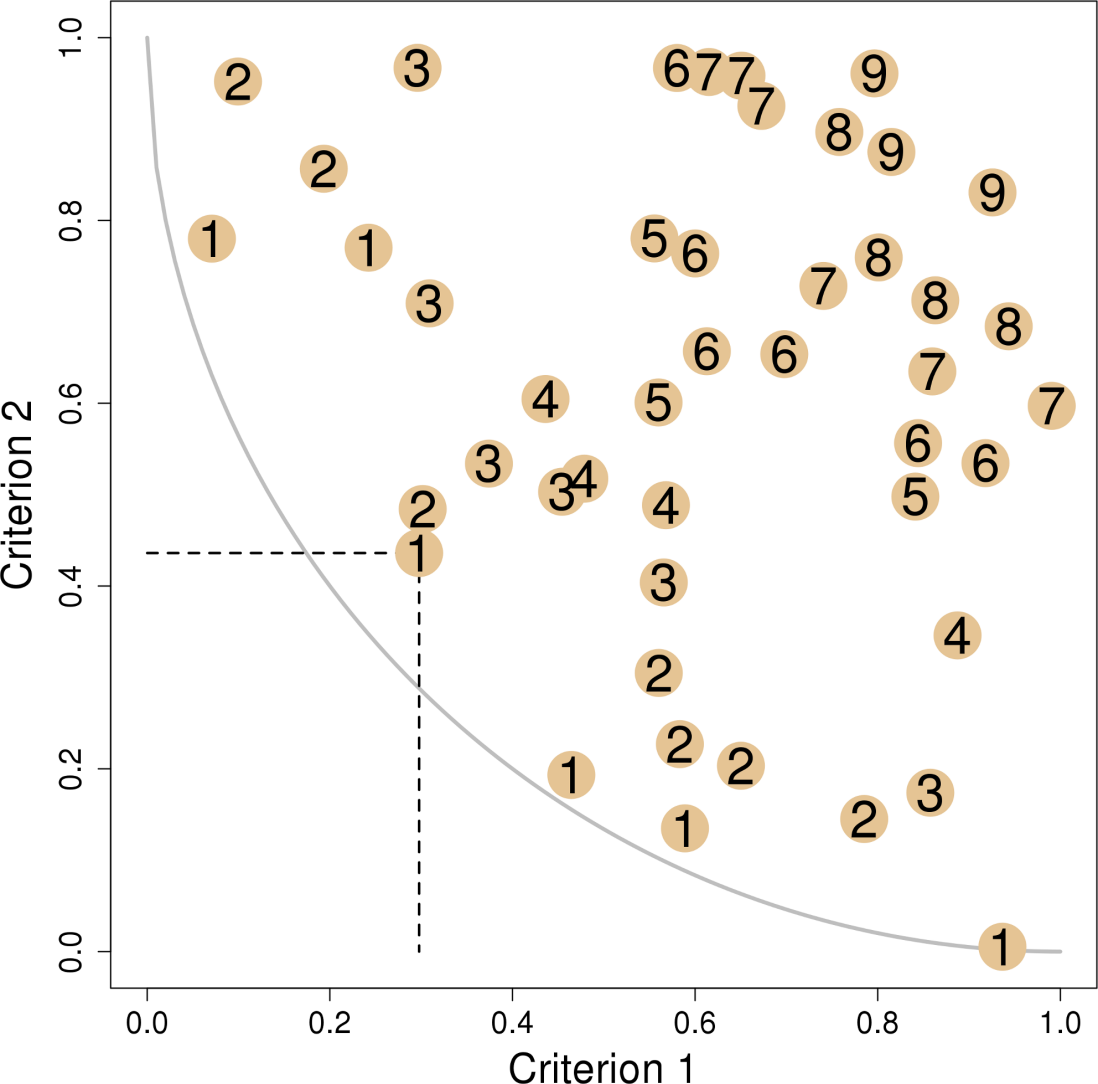
An example of Pareto ranking on two criteria. Each point represents a cell (or other spatial unit) being ranked. The first criterion is plotted on the x-axis and the second criterion is plotted on the y-axis. If no other solutions fall within a rectangle defined by a focal solution and the origin (dashed line), the focal solution is said to be non-dominated. All non-dominated solutions are given a rank of 1 and fall along a curve called the Pareto front (solid line). All rank1 solutions are removed, and then the non-dominated solutions of those that remain are given rank 2. The process is repeated until all solutions are ranked.

Pareto ranking is based on the concept of weak Pareto dominance. A vector **x**_1_ of criteria is said to dominate another vector **x**_2_ if and only if every element of **x**_1_ is less-than-or-equal-to every corresponding element of **x**_2_, and at least one element of **x**_1_ is strictly less (recall that we are treating this as a minimization problem) than the corresponding element of **x**_2_ [13] (Fig 2). If **x**_1_ weakly Pareto dominates **x**_2_, this is denoted as **x**_1_ ≺ **x**_2_. When considering many alternatives, if a vector of criteria for an alternative is not dominated by the vector of criteria for any other alternative, then it is said to be non-dominated. When plotting alternatives against each other in criteria space, the non-dominated set (NDS) falls along a Pareto front (Fig 2). Pareto ranking assigns the alternatives (grid cells) in this NDS a rank of 1; as a result, more than one alternative might be given this rank. After removing the rank-1 vectors of criteria from consideration, a new Pareto front is formed for the remaining alternatives. The non-dominated set of the remaining alternatives can be assigned a rank of 2. The Pareto ranking process continues by incrementing the current rank by 1 and assigning this rank to the NDS of the alternatives that have not been ranked; this process is iterated until all alternatives are ranked (Fig 2). The rank of the criteria vector **x**_*k*_ is denoted as *r*(**x**_*k*_). If **x**_*k*_ ≺ **x**_*l*_, then *r*(**x**_*k*_) < *r*(**x**_*l*_). In other words, an alternative with a lower Pareto rank dominates an alternative with a higher rank and, therefore, should be given a higher priority for monitoring and/or management.

We applied the Pareto ranking algorithm to each of our six different sets of criteria. We also applied it to criteria formed from the Pareto ranks based on two of the six decision matrices of criteria, which we refer to as “composite Pareto ranks” denoted as *r*[*r*(**F**′ | **S**′) | *r*(**H**′ | **S**′)], *r*[*r*(**F**′ | **G**′) | *r*(**H**′ | **G**′)], and *r*[*r*(**F**′ | **S**′ | **G**′) | *r*(**H**′ | **S**′ | **G**′)]. We included this application of composite Pareto ranking to increase the potential range of rankings (i.e. the maximum rank) because as the number of criteria considered in Pareto ranking increases, more spatial units (e.g. grid cells) will have lower ranks, making it more difficult for one vector of criteria associated with the spatial unit to dominate another in all criteria. Additional details on composite Pareto ranking are given in S2 Appendix.

The correlation structure among criteria has an influence on the Pareto ranking process. Simulation studies that we conducted indicate that the maximum rank assigned to any vector of criteria in a set of vectors depends on the number of criteria in the vectors, correlations among the criteria, and the number of units being ranked (S2 Appendix). While independence among criteria is not a requirement for Pareto ranking, as the correlation among criteria increases from -1.0 to 1.0, the range of the Pareto ranks also tends to increase. This implies that when criteria become increasingly negatively correlated the range of ranks decreases, resulting in more spatial units being assigned to each rank. If there are few ranks to which a large number of spatial units must be assigned, decision-makers may have to perform a second-level decision analysis to select the final set of prioritized units. As a simple example, in Fig 2 six units were assigned a rank of 1 and seven were assigned a rank of 2. If the decision target was to select ten units, the six rank-1 units would be chosen first, but then another decision rule would be needed to select only four of the seven rank-2 units. If those criteria were strongly negatively correlated, then there could easily have more than ten rank-1 units in this example. Therefore, we also describe the correlation among the attributes (which applies to criteria, since the attributes were transformed into criteria using a linear value function).

We based our implementation in the R/C++ [41, 42] programming languages on the algorithm given by Xiao et al. [13], which we optimized and modified to account for vectors equal in all criteria (all code is available in S1 File). Once all alternative grid cells were ranked, we mapped the cell rankings for each decision matrix and summarized the results by climate zone and conservation status. A more complete set of output, including data tables with criteria and ranks for each grid cell can be produced for regional resource managers and other decision-makers.

## Results

### Spatial distribution of attributes

**Fig 3:**
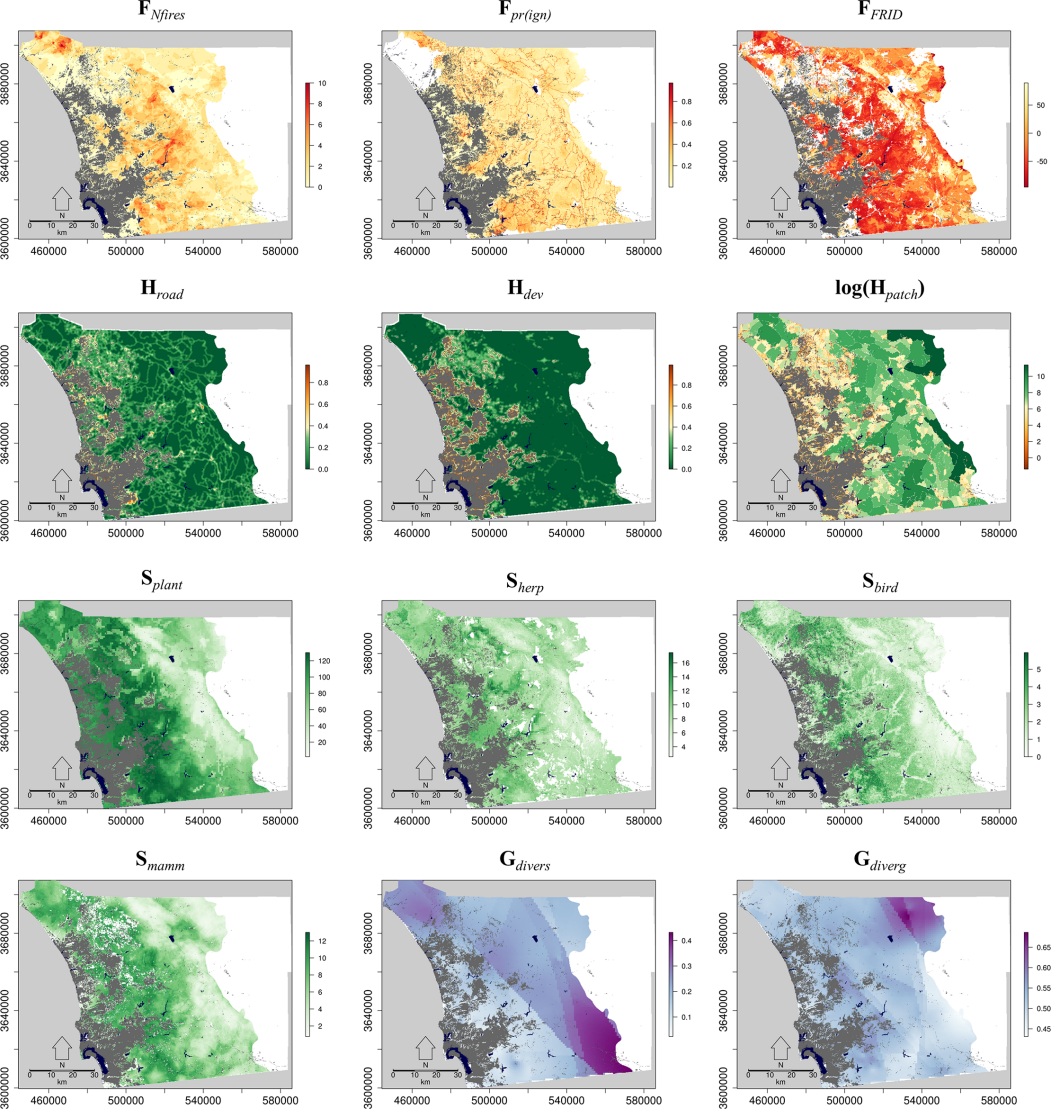
Maps of ranking criteria: Number of fires (F_*Nfires*_), predicted ignition probability in patches (F_*pr(ign)*_), mean fire return interval departure (F_*FRID*_), all road density within 500 meters (H_*road*_), weighted development density within 500 meters (H_*dev*_), log patch area in hectares (H_*patch*_), index of plant biodiversity (S_*plant*_), index of herpetofauna biodiversity (S_*herp*_), index of bird biodiversity (S_*bird*_), index of mammal biodiversity (S_*mamm*_), genetic diversity (G_*divers*_), and genetic divergence (G_*diverg*_). Darker colors represent higher levels of a particular criterion. For fragmentation criteria, fragmentation increases as the color transitions from dark green to brown. The criteria are described in Tables 1 and 2.

**Fig 4:**
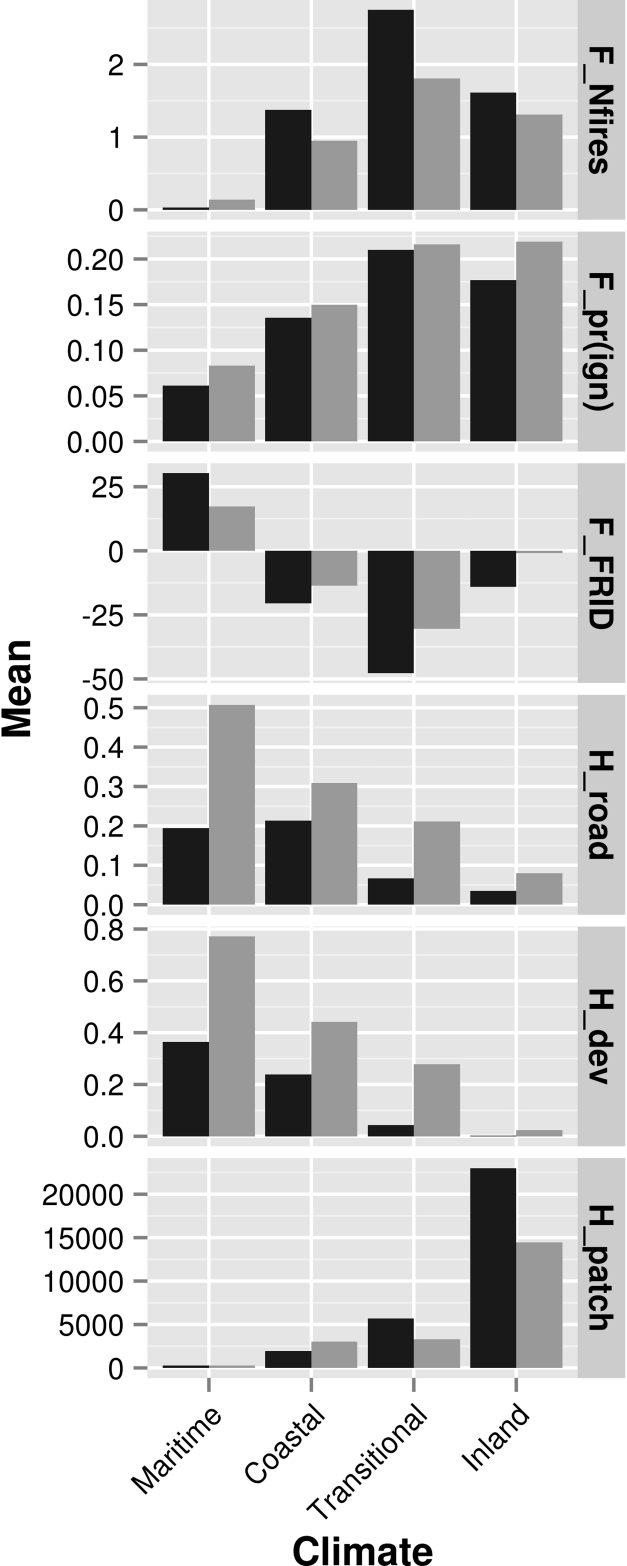
The mean value of threat criteria by climate zone and conservation status. Criteria are described in Table 1. Conserved lands are shown in dark gray and non-conserved lands are shown in light gray.

All three fire-related criteria indicated the threat of fire increases eastward from the coast until it reaches its peak in the transitional climate zone, then decreases in the mountainous inland climate zone (Figs 3 and 4). Conserved lands tended to have more fires and a lower FRID, which indicated more frequent historic fires relative to the estimated pre-European frequency (Fig 4). In contrast, FRID tended to be positive in the maritime climate zone, suggesting less frequent fires compared to the pre-historic regime (Fig 4). However, predicted probability of large-fire ignition tended to be higher in non-conserved land. This is due in part to the importance of development-related predictors in the ignition models [17]. As expected, mean local road and development density was highest in the maritime climate zone and declined eastward through the coastal, transitional, and inland climate zones (Figs 3 and 4). Similarly, fragment (patch) area was smallest near the coast and increased as we moved eastward. The road and development density was lower and patch sizes were larger in conserved lands than in non-conserved lands when averaged within climate zones.

**Fig 5:**
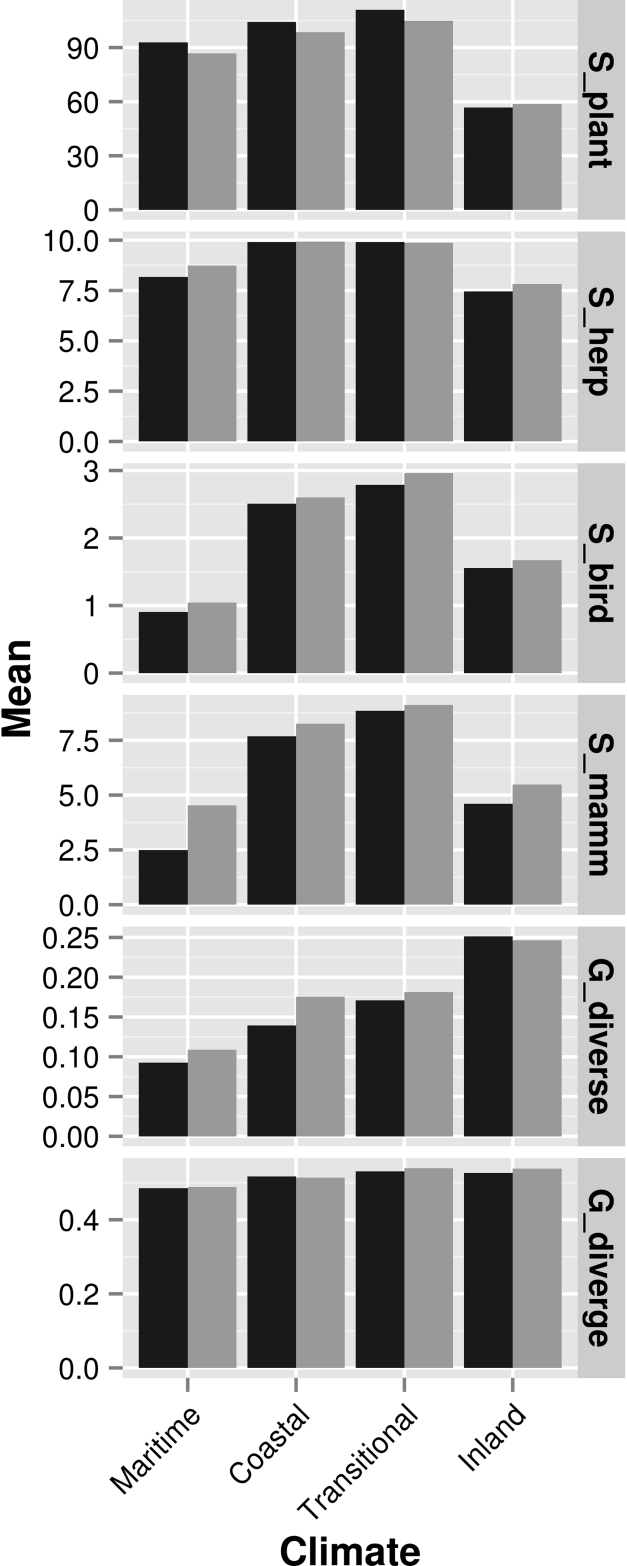
The mean value of biodiversity criteria by climate zone and conservation status. Criteria are described in Table 2. Conserved lands are shown in dark gray and non-conserved lands are shown in light gray.

Biodiversity variables for all four taxonomic groups showed a similar pattern to fire variables; that is, diversity increased eastward from the coast through the maritime, coastal, and transitional climates zones, but then declined in the inland zone (Figs 3 and 5). Herpetofauna diversity was more uniform across all climate zones compared to other taxa (Fig 5). Within each zone, there were distinct patches of higher predicted diversity for each taxon (Fig 3). Somewhat unexpectedly, mean predicted species diversity was slightly, but consistently, higher in non-conserved lands compared to conserved lands in all climate zones for all taxonomic groups except plants (Fig 5). Mean predicted genetic diversity increased from the coast eastward within climate zones (Fig 5). The highest predicted diversity was in the southeast corner of San Diego County (Fig 3). The mean predicted genetic divergence was fairly uniform across climate zones (Fig 5) but had a distinct area of higher divergence in the northeast corner of San Diego County (Fig 3).

### Correlations between attributes

Within fire threat criteria, **F**_*FRID*_ was strongly negatively correlated with **F**_*Nfires*_ (see S3 Appendix for all correlations). Development density (**H**_*dev*_) and road density (**H**_*road*_) were strongly positively correlated. Fire and habitat fragmentation criteria did not show a strong correlation. All predicted species diversity criteria were strongly positively correlated. In contrast, the two genetic criteria were negatively correlated. Genetic diversity was also negatively correlated with all species richness criteria. Number of fires (**F**_*Nfires*_) was positively correlated with species richness criteria and, as expected given the relation between **F**_*Nfires*_ and **F**_*FRID*_, **F**_*FRID*_ was negatively correlated with the species richness criteria.

Fire and habitat fragmentation were only weakly correlated in space, with more fragmentation in the maritime and coastal climate zones and greater threat of fire in the transitional and inland climate zones (Figs 3 and 4). Predicted species diversity was highest in the transitional climate zone for all four taxa, but plant richness was also high in the maritime and coastal zones. Mammal diversity was also moderate in the coastal and inland zones, and herpetofauna diversity was more uniformly distributed across the study area. Genetic criteria had a much different spatial distribution than the other criteria: predicted genetic diversity was highest in the southern part of the inland climate zone while predicted genetic divergence was highest in the northern part of the inland zone. As a result, predicted genetic diversity was negatively correlated with development and road density as well as predicted plant richness.

### Cell ranks

**Fig 6:**
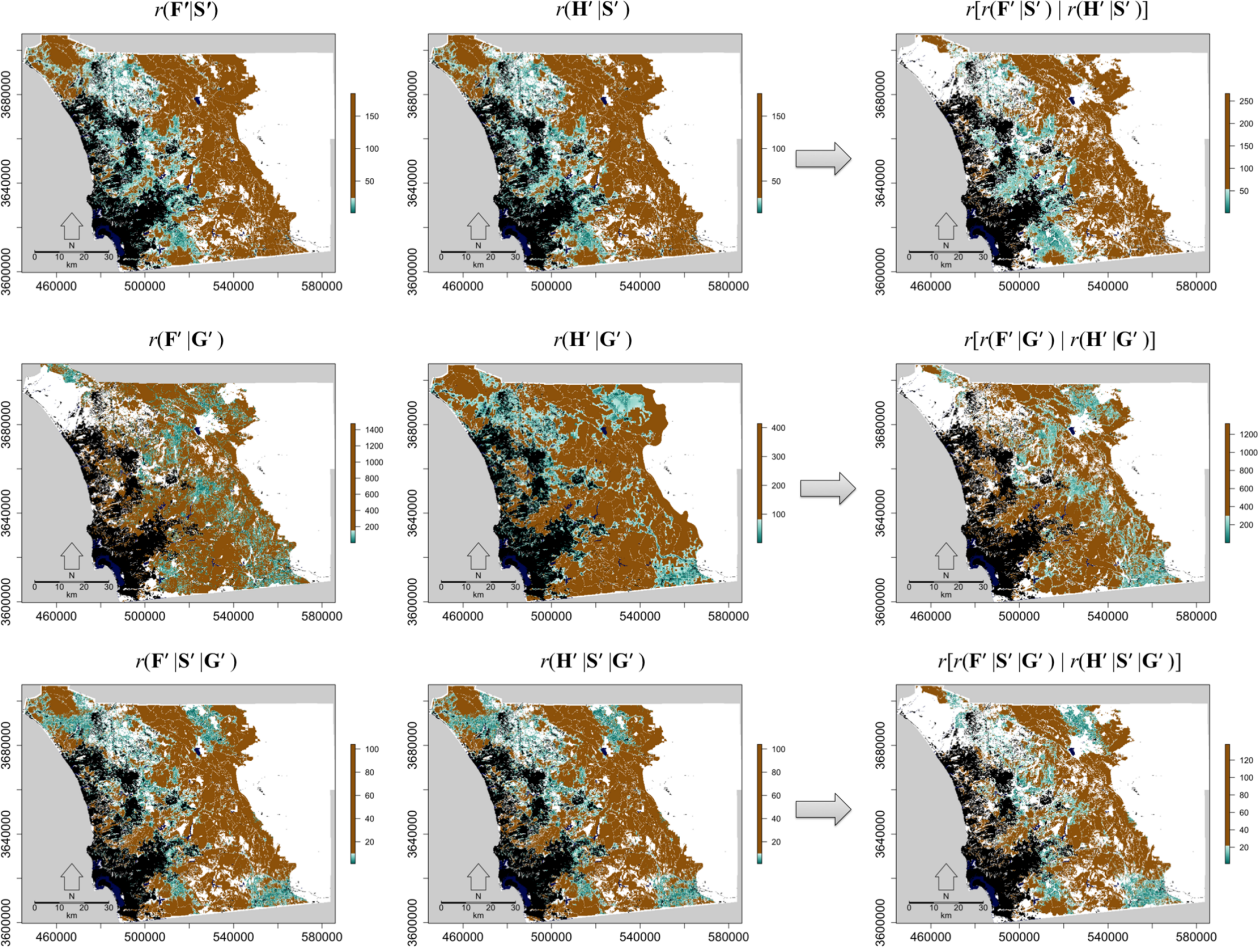
Maps of cell Pareto ranks for each decision matrix. The decision matrix used is indicated above each map (**F**′, **H**′, **S**′, and **G**′ denote the matrix of fire, habitat fragmentation, species, and genetic criteria, respectively). The right-most column shows composite ranks which are Pareto ranks of the Pareto ranks based on the two decision matrices to the left. All ranks at or below the 25^th^ percentile transition from red to yellow as the rank increases. All ranks above the 25^th^ percentile are shown in green.

**Fig 7:**
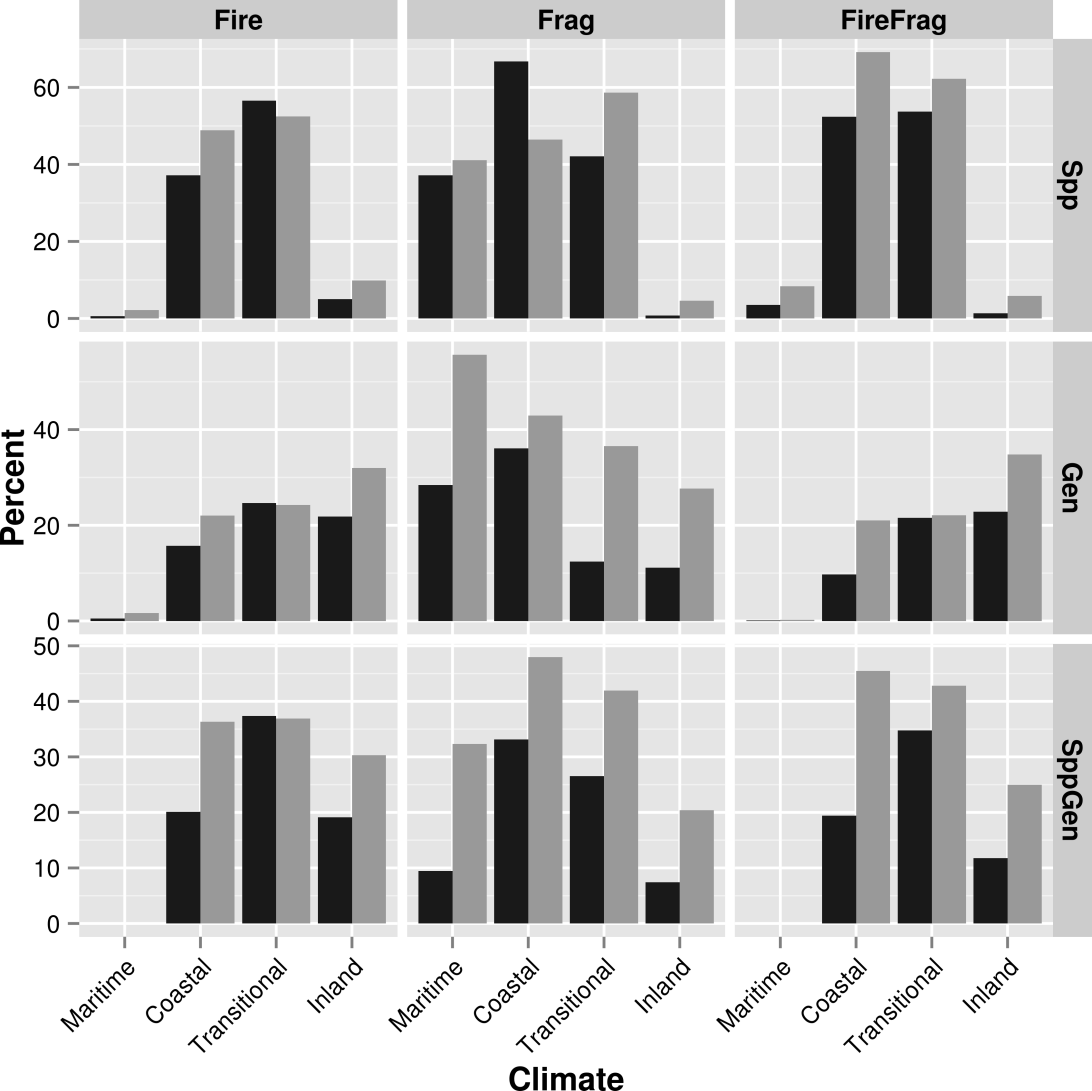
A summary of the percent of cells within each climate zone and conservation status that pass our threshold for increased fire monitoring and management for each set of ranking criteria. We set the threshold for ranks at or below the 25th percentile across all cells for each set of criteria. Each panel corresponds to a different set of criteria composed of threat-related criteria (in columns) and biodiversity-related criteria (in rows). Percentages are shown separately for conserved lands (dark gray) and non-conserved lands (light gray). These percentages indicate where conservation efforts should be focused by climate zone and conservation status (e.g. a value of 50 percent would indicate half the cells for that zone and status pass our 25^th^ percentile threshold).

**Fig 8:**
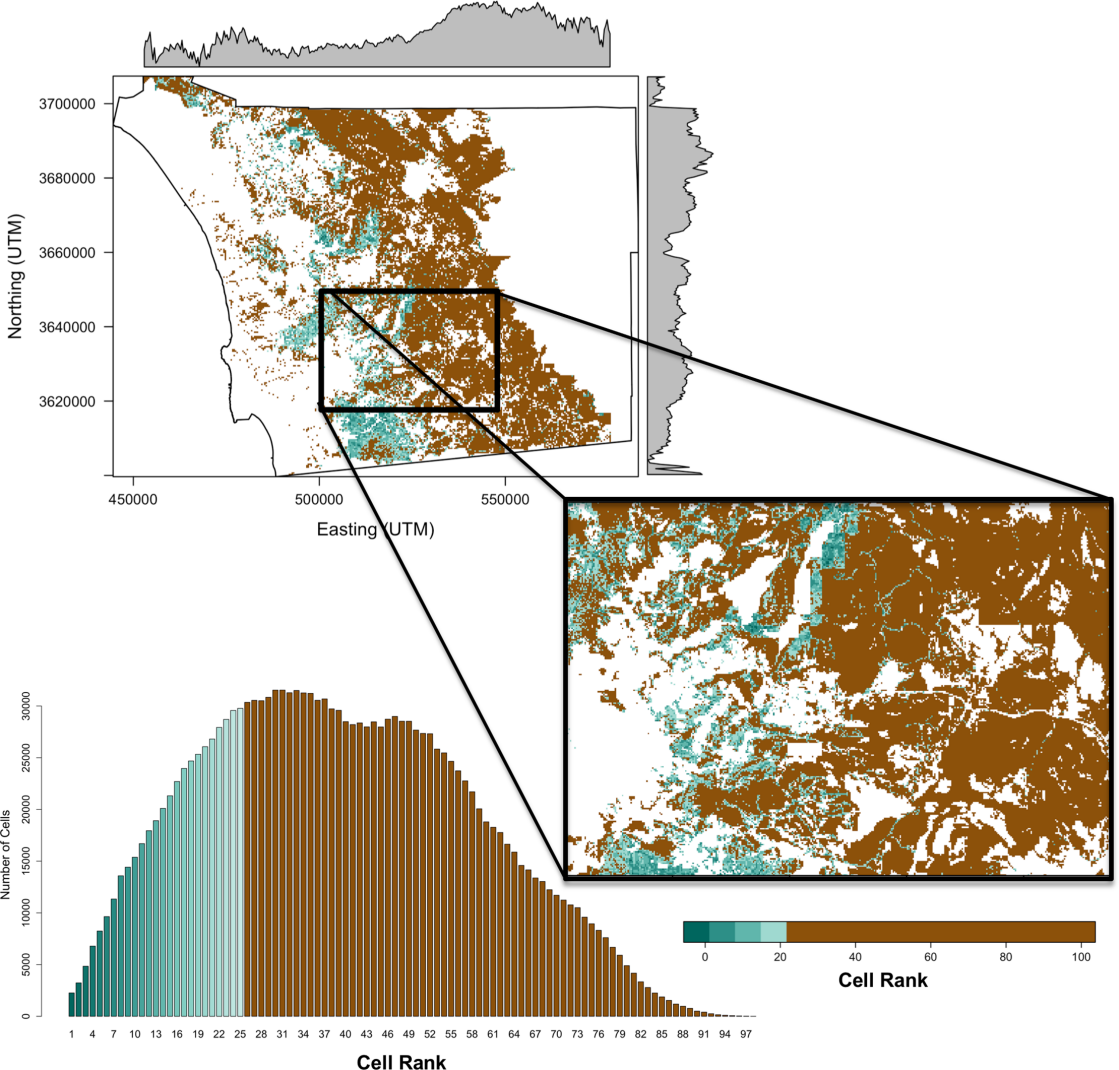
A detailed view of the rankings for the fire (F) and species (S) criteria. Ranks are shown for the entire County (top left). The median grid cell ranks for each y-coordinate (northing) is shown on the right margin, and the median grid cell ranks for each x-coordinate (easting) is shown on the top margin. Note that a lower median rank indicates a higher priority. An enlarged region along a gradient of ranks is shown on the right. A bar plot of the cell ranks, colored in the same way as in the maps, is shown on the lower left. All ranks at or below the 25^th^ percentile transition from red to yellow as the rank increases. All ranks above the 25^th^ percentile are shown in green.

We mapped the Pareto ranks for each of the six decision matrices and the three composite ranks (Fig 6) and summarized results within climate zones and conservation status by calculating the proportion of grid cells with a rank equal-to-or-less-than the 25^th^ percentile (Fig 7). For most criteria, cells in the transitional and coastal climate zones, particularly for non-conserved lands, had high proportions of cells at or below the 25^th^ percentile, indicating that these areas are higher priority for monitoring and management (Fig 7). Many of the lower-ranking grid cells, for most criteria sets, fell along the eastern edge of major urban areas (Fig 6). Fire threats overlapped considerably in areas with high biodiversity, particularly in the central and southern part of the transitional climate zone (Fig 3). Hence, more cells were given a higher priority (lower rank) in this region indicating these areas should be important to consider for fire management (Fig 6). The maritime climate zone generally had a low proportion of cell ranks at or below the 25^th^ percentile, except for decision matrices [**H**′ | **S**′] and [**H**′ | **G**′] that included fragmentation and either species richness or genetic criteria, respectively (Fig 7). Unless genetic criteria were included, the mountainous inland climate zone had a low proportion of cells that ranked at or below the 25^th^ percentile (Fig 7). Usually, even when genetic criteria were included, the inland climate zone had a lower proportion of cells that ranked at or below the 25^th^ percentile, compared to the coastal and transitional zones. Although we have described the general spatial patterns of cell ranks for the region, detailed maps needed for conservation monitoring and management of specific areas can be provided to land managers (Fig 8).

## Discussion

Our objective was to demonstrate a method for prioritizing areas for conservation monitoring and management based on Pareto ranking. We used biodiversity-related attributes, but other attributes (e.g. distributions of endemic species, patch edge versus core, or metrics of landscape connectivity) can be used. Our overall approach to prioritizing areas for conservation monitoring and management consisted of four steps: (1) define the spatial units to be prioritized, (2) develop attributes describing biodiversity and threats associated with each spatial unit and combine species distribution models (SDMs) to produce attributes describing species diversity within taxonomic groups, (3) apply value functions to each attribute to produce criteria reflecting preferences, and (4) apply Pareto ranking to the criteria to prioritize the spatial units. Although we used grid cells to define our spatial units, the approach could also be applied to points, lines, or polygons representing landscape features. When we combined SDMs within each taxonomic group, we weighted all species equally; however, unequal weights could be applied based on the importance of each species to the prioritization problem.

We applied this method to prioritize areas of San Diego County, California. By identifying areas with high biodiversity and high threats of fire, we can design future studies to improve our understanding of this disturbance threat on conservation assets. Furthermore, by knowing where high biodiversity and threats of fire and habitat fragmentation all co-occur, we have identified places that could warrant additional conservation land acquisition and increased protection from wildfire. For example, land managers may want increased protection for areas with high genetic biodiversity and threat of wildfire so that we can maintain reservoirs of genetic diversity to facilitate adaptation to climate change. Data layers with the Pareto ranking results can be used by local land managers for planning within the specific habitat preserves under their supervision.

When summarized, the coastal and transitional climate zones are under threat from both habitat fragmentation and wildfire (Fig 4). These climate zones also had the highest predicted species diversity for plants, herpetofauna, birds, and mammals (Fig 5). In contrast, the areas with the highest predicted genetic diversity and divergence were in the inland climate zone (Figs 4 and 5), where threats from fire and fragmentation were lower. Because the combined effect of fragmentation and fire was highest in the coastal and transitional climate zones where species richness is also highest, most of the lower-ranked grid cells occurred in these zones and therefore, these areas could be a higher priority for monitoring and management.

Although our results suggest where fire and fragmentation are more likely to impact biodiversity, they do not address which specific impacts can be expected. Negative impacts of fragmentation on plants, birds, mammals, and other taxa are well understood in our study area [43–47], but effects of increasing fire frequency on biodiversity are less clear. Frequent fires may induce vegetation type conversion from shrub to grassland, which may further contribute to altered fire regimes [21]. This type conversion can adversely affect animal communities that depend on native vegetation. Based on previous studies of post-fire communities of herpetofauna and small mammals [22, 23, 48], closed habitat specialists (i.e. those that prefer dense vegetation or other structural cover) show a decline while generalists and open habitat specialists tend to increase in relative abundance in the decade immediately following a fire. However, for many faunal species, the long-term impacts of large fire events or changes in fire regimes are unclear. By identifying areas with high fire threat and high biodiversity, researchers might design better studies to reveal the impact of wildfire and habitat fragmentation on animal communities, including pre-fire and post-fire comparisons.

Wildfire and habitat fragmentation are not completely independent factors. For example, increased human-caused fire ignitions and increased fire suppression are both more likely near the more fragmented urban and other human-occupied areas [49–51]. Conversely, reduced structural connectivity of vegetation as a result of fragmentation can inhibit the spread of wildfire in some areas [52]. Habitat fragmentation, particularly from roads, fuel breaks, and trail edges, may also increase the abundance of non-native plant species that may colonize areas after fires [53–56]. While fragmentation or fire alone may pose a risk to species persistence, the combined effect of these threats may be more detrimental to some species. Where altered fire regimes and habitat fragmentation co-occur, we would expect habitat fragmentation to reduce the ability of some species to find refuge and recolonize areas following fires. On the other hand, if fragmentation prevents the spread of fire to some habitat patches, this can potentially shield refugia for species that can persist in the isolated patches.

Several trends may alter future fire regimes and the distribution of species on the landscape. First, the San Diego Association of Governments has projected 140 percent increase in human population in San Diego County by 2050; therefore, we can expect further development (urbanization, agriculture, roads, power transmission lines, etc.) and hence increased habitat fragmentation, particularly in the coastal and transitional climate zones. This projected increase in human population and associated development may lead to increased fire ignitions [17]. Second, climate change may affect abiotic conditions such as temperature and moisture which can alter fire regimes. Climate change may also cause shifts in species distributions and phenology [57–59], with feedbacks to the spatial and temporal pattern of fire risk [60].

When applying Pareto ranking or any type of decision optimization, it is important that decision makers establish a clear link between ranking, management actions, and conservation goals. We highlight two related challenges of using the Pareto ranking approach for conservation planning. First, negative correlations and larger numbers of criteria can reduce the range of ranks assigned to units, making the prioritization less fine-grained (categorically detailed). As the number of criteria increase, the probability of one vector of criteria dominating another will decrease. When two criteria are positively correlated, then one spatial unit is more likely to dominate or be dominated in both criteria. Thus, a criterion that is strongly positively correlated with another criterion that is already used will have less effect on the overall ranking process. In contrast, with criteria that are negatively correlated, one spatial unit is less likely to dominate, making it more difficult for the spatial unit to dominate the other in all criteria. Thus, having strongly negatively correlated criteria reduces the range of ranks assigned to spatial units and increases the number of units within each rank.

Secondly, additional prioritization may need to be applied to spatial units that have been assigned the same rank. Often, it is not necessary for the decision analysis to assign a unique rank to every unit, however; instead, the number of units may just need to be reduced to a manageable number. In this case, Pareto ranking can be (and often has been) used as a standalone method for decision analysis. Alternatively, if a unique rank is desired for each unit, at least among the most favorable units from which the final selection will be made, Pareto ranking may be used as a screening method to remove the higher-ranking units (whose criteria are dominated by the lower ranking units and should never be chosen). After screening out most units by Pareto ranking, another decision analysis technique may be applied to uniquely rank the remaining units. However, as long as lower-ranked alternatives are selected over higher-ranked ones, additional prioritization within ranks will not preclude non-dominated alternatives from being selected over dominated ones. Additional prioritization within ranks could actually represent an advantage of the approach, allowing managers to use their expert knowledge to further prioritize alternatives within the constraints of Pareto ranking.

The Pareto ranking approach also has several important advantages when applied to conservation and management. Attributes do not need to be converted to criteria on the same scale (e.g. a preference scale) because different criteria are not added or compared. Comparisons are only made within criteria during Pareto ranking. This property of Pareto ranking can be particularly beneficial because it is not necessary to assign an economic value to conservation considerations. In addition, because criteria are not added, a high preference for one (or more) criteria does not compensate for low preferences in the others. Second, while decision makers must still select the attributes/criteria that will be used in the Pareto ranking algorithm, there is no need to select weights as part of the decision-making process. In comparison to the commonly-used additive MAVT approach, weighting criteria has no effect on the Pareto ranking process. For example, suppose weights are assigned to each criterion as we do in the additive MAVT approach. In this case, a total value can be assigned to each alternative using $s_{k}=\sum_{m=1}^{M}w_{m}f_{m}(a_{k,m})$ and then ranked on *s*_*k*_ [9]. Pareto ranking is then applied, comparing each criterion individually in pairs of vectors of criteria for each alternative (labeled here as vector *k* and *l*) to determine if *w*_*m*_*f*_*m*_(*a*_*k*,*m*_) ≤ *w*_*m*_*f*_*m*_(*a*_*l*,*m*_), which is equivalent to *f*_*m*_(*a*_*k*,*m*_) ≤ *f*_*m*_(*x*_*l*,*m*_) for any *w*_*m*_ > 0. Further, after Pareto ranking, any lower-ranking alternative will always beat higher-ranking alternative if the weighted sum approach to MVAT was used for additional screening regardless of the weights selected (assuming positive weights and criteria). Therefore, assigning weights to criteria and disagreements about the weights are irrelevant in Pareto ranking, because Pareto ranks reflect subjective values.

We have several directions for future work. With respect to the Pareto ranking algorithm itself, we are interested in further investigations in two areas. First, methods for reducing dimensionality, such as Principle Components Analysis (PCA), may be helpful in producing sets of independent criteria for ranking. However, the use of these methods in conjunction with Pareto ranking has not, to our knowledge, been studied. Second, while our analysis involves spatial data as inputs to the ranking process and the results are spatially referenced, it does not take into account spatial relationships among the units being ranked. Regarding our application of Pareto ranking, we have four foci of future work. First, the criteria used in decision making are dynamic and we plan to continue work on developing spatial models for them. The second focus is to develop a deeper understanding of the impacts of fire and/or fragmentation on individual species and species richness over different time scales. Understanding temporal impacts, combined with prioritizing grid cells based on the interaction of biodiversity, fire, and fragmentation will allow us to understand better the risks faced by biodiversity in our region. Third, if predictions for fire, fragmentation, and biodiversity (e.g. species distributions and genetic diversity) are available under climate change and urbanization scenarios [61], we can apply Pareto ranking to future scenarios. Finally, the rich biodiversity of Mediterranean-type ecosystems in areas undergoing rapid climate change (i.e. in southern California) is challenged by existing and future habitat fragmentation, potentially changing fire and hydrological regimes, climate change, invasive species, and introduced pathogens. If these additional threats are quantified or predicted spatially, they can be added to future Pareto ranking schemes to provide a more complete assessment.

We successfully used Pareto ranking to prioritize a large number of spatial units in San Diego County, California, USA based on criteria related to species and genetic biodiversity and threat of wildfire and habitat fragmentation. Although Pareto ranking has seen little use in conservation science, it is broadly applicable and has several advantages over commonly used methods. Spatial prioritization methods, such as the one presented here, can help researchers and resource managers focus limited resources more effectively.

## Supporting information

S1 File R and C++ Code for pareto ranking. (ZIP)

S1 Appendix Additional information on threat and biodiversity criteria development. (DOCX)

S2 Appendix Illustrative example of pareto ranking. (DOCX)

S3 Appendix Summary statistics and correlation coefficients for criteria. (DOCX)
