# Supplementary material for: Prioritizing conserved areas threatened by wildfire and fragmentation for monitoring and management
Source: PLoS One. 2018 Sep 7;13(9):e0200203. doi: 10.1371/journal.pone.0200203 (PMC6128460; doi:10.1371/journal.pone.0200203)
Supplement: S1 Appendix — (DOCX) [file pone.0200203.s002.docx]

**S1 Appendix: Additional Information on Threat and Biodiversity Criteria Development**

We developed spatial criteria related to indicators of high biodiversity (predicted plant, herpetofauna, and mammal species richness and predicted genetic diversity and divergence) and indicators of threat to that biodiversity due to fire and habitat fragmentation. Criteria were computed for each 50-meter grid cell in the study area. The cell-level variables were then aggregated to the analysis patch-level by taking the mean of the values of the cells within each patch.

**Fire threats**

We used three fire-related criteria: the number of historic fires, which indicates overall fire frequency over the last century; mean predicted probability of the ignition of large fires, which indicates the potential for new fires being started; and, mean fire return interval departure, which indicates the change of estimated fire inter-event times (years between fires) for the historic time period compared to the pre-settlement times (Safford et al. 2011).

Number of Historic Fires. We used CalFire fire perimeters for 1878 – 2012 as the basis for describing fire history in San Diego County (fire perimeters downloaded from http://frap.fire.ca.gov/data/frapgisdata-subset.php). The earliest recorded fire perimeter for San Diego County was in 1910. The perimeters were compiled from the U.S. Forest Service (USFS), Bureau of Land Management (BLM), the National Park Service (NPS), and local jurisdictions. Federal Agencies (NPS, BLM, and USFS) reported fires that were ≥ 10 acres. CAL FIRE reported timber fires ≥ 10 acres, brush fires ≥ 50 acres, grass ≥ 300 acres, and fires that destroyed ≥ 3 residential or commercial structures. Using the raster package in R (Hijmans 2013), we rasterized every fire perimeter polygon at a 50 m resolution, and constructed a table to index the fire rasters by year and other attributes. We then added the fire perimeter rasters to compute the total number of fires per cell from 1910 to 2012.

Probability of Ignition. In addition to total number of fires, we used predictions for the probability of ignition of large fires that were made using point data for ignition locations for fires (Syphard and Keeley 2015).

Fire Return Interval Departure. Finally, we included USFS estimates of fire return interval departure (FRID; ([Safford et al. 2011](#_ENREF_50))). We used MedianFreqDep, which is a measure of the departure of the current fire return interval (CurrentFRI) from the median reference fire return interval (MedianRefFRI). CurrentFRI is calculated by dividing the number of years in the fire record by one plus the number of fires (based on the CAL FIRE perimeters) that have occurred in the record at each location (that is, CurrentFRI = Years/[NumberOfFires+1]). MedianRefFRI are averages of the median of pre-Euroamerican FRI intervals for major vegetation types (based on CalVeg) taken from an exhaustive review of the published and unpublished literature. It is calculated as:

$$\text{MedianFreqDep}=\left\{\begin{matrix} 100\times\left(1-\frac{\text{MedianRefFRI}}{\text{CurrentFRI}}\right) \\ -100\times\left(1-\frac{\text{CurrentFRI}}{\text{MedianRefFRI}}\right)\text{ } \end{matrix}\right.\begin{matrix} \text{if CurrentFRI > MedianRefFRI} \\ \text{otherwise} \end{matrix}$$

Thus, a negative value for MedianFrepDep indicates that intervals between recorded fires in the last century are shorter than they are believed to be in the pre-Euroamerican fire regime. In other words, fires are believed to be more frequent than they once were.

**Road and development impact**

We used development density (dataset, CalVeg), the density of all roads (dataset, SANDAG), and fragment area as indicators of habitat fragmentation.

Development Density. We based a criterion for development density on the CalVeg land cover data for urban areas as (WHRTYPE = URB) and agricultural areas that included cropland, orchards, eucalyptus groves, and pasture (WHRTYPE = CRP, DOR, EOR, EUC, PAS, or VIN). For each grid cell, we described the impact of urban and agricultural development as [(number of urban cells within 500 m) + 0.3*(number of agriculture cells within 500m)]/(total number of cells within 500 m). The radius of 500 meters was selected based on Hunter et al. (2003), and the weight of 0.3 for agricultural land was selected based on expert opinion. Thus, each cell is assigned a value between 0.0 and 1.0, where larger values indicate a higher proportion of development surrounding the cell. When aggregated to the patch level, patches bordered by developed areas with have a larger value, particularly if they are small and/or have a large perimeter-to-area ratio.

Road Density. For each grid cell, we calculated a value indicating the impact of all roads on each cell (number of cells intersected by a road within 500 m)/(total number of cells within 500 m) using rasterized SANDAG ‘all road' line data (SANDAG). Like development impact, values range from 0.0 to 1.0 and will have higher mean values in patches bordered by roads, particularly if they are small and/or have a large perimeter-to-area ratio.

Fragment Area. Area (in hectares) was calculated as an attribute for patches of continuous road-free, non-urban areas. In this case, SANDAG ‘all roads’ data were used to delineate road-free patches instead of the SANDAG ‘major roads’ line data that were used to delineated patches for analysis. The fragment area of the patch was assigned to each grid cell in the fragment.

**Biodiversity Indices**

Using available species distribution models (SDMs) we developed criteria for an estimated index of biodiversity based on 138 plant species, 29 reptile and amphibian (herpetofauna) species, 6 bird and 18 mammal species (S1 Table). We describe each of these datasets in more detail. A common pattern among these criteria is that predictions were made for all species for which sufficient data could be acquired, and therefore not total taxonomic diversity.

Plants. We used SDMs for 138 plant species that were developed by The Nature Conservancy using a maximum entropy (MaxEnt) approach to investigate climate change impacts under alternative scenarios (Phillips et al. 2006, Principe et al. 2013). Life forms of the species included in our analysis included ferns, grasses, herbs, vines, shrubs, trees, cacti and other succulents and included facultative seeder, fire follower, obligate resprouter, and obligate seeder fire adaptations (http://www.calflora.org, Halsey 2005). As such, the species used were a good representation of the region’s over 1500 native plant species. Species observations from > 4500 locations from studies conducted in San Diego County by U.S. Geological Survey, U.S. Fish & Wildlife Service, the San Diego Management and Monitoring Program, California Department of Fish and Game, and AECOM were used. Species were included if they were present in at least 30 locations and in two or more of the datasets from these studies. The continuous probability of presence on the interval from 0.0 to 1.0 was converted to binary scores (1 = suitable, 0 = unsuitable) using thresholds that maximized the recovery of both true positives and true negatives for each species in testing data ([Liu et al. 2005](#_ENREF_33)). The binary scores for the 138 plant species were summed to compute predicted plant species richness (species are listed in S1 Table).

Reptiles and Amphibians (herpetofauna). We used SDMs developed by Franklin et al. (2009) for 24 reptiles and five amphibians. For each species both a generalized additive model (GAM; Wood 2006) and Random Forest (RF; Cutler et al. 2007) approach was used. Species data were collected between 1995 and 2008 by Fisher et al. (Fisher et al. 2008) as part of ongoing herpetofauna pitfall surveys. Data from 591 pitfall arrays were used, and species distribution models for species that were detected in > 5 percent of the arrays were developed. Covariates developed during previous research (Franklin et al. 2009, Syphard and Franklin 2010), including mean January minimum temperature, mean July maximum temperature, mean annual precipitation, mean summer solstice solar radiation, slope gradient, soil order, available water capacity, soil depth, soil pH (STATSGO), and vegetation (CalVeg), were used as in the GAMs and RF models. Model prediction performance was evaluated using the area under the curve (AUC) for receiver operating characteristic (ROC) plots (Hanley and McNeil 1982). The model (GAM versus RF) with the highest AUC was used as the final model to predict the distribution of each species. The probability of presence for the 29 species was summed to yield predicted herpetofauna species richness (S1 Table).

Mammals. The San Diego Natural History Museum and USGS developed the species occurrence data and distribution maps for mammals as part of a study funded by San Diego County. Occurrence data were compiled from electronic searches of museum collections with large holdings of mammals (American Museum of Natural History, New York; Chicago Field Museum; Museum of Southwestern Biology, University of New Mexico; Museum of Vertebrate Zoology, Berkeley; Natural History Museum of LA County; the San Diego Natural History Museum; University of Kansas, Mammal Collection; and the Smithsonian, Washington D. C.). In addition, we collected data from local sources such as the Anza Borrego State Park, our own trapping records, data from colleagues, and data from a literature search (including academic and government documents) of studies, surveys, or monitoring performed in San Diego County.

Occurrence data were quality controlled using a variety of methods. First, when appropriate, we corrected locational errors. Some points were outside of the county, mapped in the Pacific Ocean, or placed species well outside their known ranges (e.g. a desert species in coniferous forest). The majority of these cases were caused by database entry errors associated with reversed cardinal directions. When sufficient information was available, we moved these points; otherwise they were not used. In total, the database contained 29,152 points locations for 98 species.

Raw occurrence data were filtered in two ways. First, we deleted historic records that were now located in urban areas. We did this because it was unlikely the species currently occurred in these places, and our ecogeographic variables used for distribution modeling represented the modern landscape, not the historic. Second, we combined multiple occurrences at a single location. In some cases, our data came from trapping grids and so a single species was recorded many times but at slightly different locations. Leaving all occurrences at this small geographic area biased results in preliminary runs of Biomapper, so we reduced these multiple records to a single record, which was a coarse adjustment for sampling effort. After these steps, any species with more than 40 unique locations was modeled (a total of 38 species).

We compiled county-wide data on soils, vegetation, water bodies including marshes and wetlands, precipitation, temperature, roads, slope, elevation, and aspect to develop 57 ecogeographic variables used as predictors of species distributions in Biomapper. For vegetation, soils, and water bodies, we developed two variables for each variable type. First, using a moving window, we estimated the percent of pixels of this type in a 3km circle around a pixel and assigned this value to the pixel. Second, we estimated the nearest distance from a pixel to a particular variable type and assigned this value to the pixel. Elevation, max and min temperatures, and precipitations were directly taken from the underlying data sources.

We performed distribution modeling using ecological niche factor analysis, implemented in Biomapper. Biomapper uses presence-only data, and ecogeographic variables to compute factors (similar to those calculated in principal components analysis) that explain successive levels of variation in the species distribution. Each factor is independent and the first factor (marginality) describes the difference between the species mean (i.e. locations where the species was found) and that of mean of the study area. The remaining factors (specialization factors) describe how specialized the species is relative to the available range of variation in study area. We used the ‘medians algorithm’ in Biomapper to compute a habitat suitability score (ranging from 0-100) for each species based on the ecological niche factor analysis ([Hirzel et al. 2002](#_ENREF_29)) and evaluated the quality of the model using summary output information relating to the levels of variation explained, warnings associated with large eigenvalues, and cross validation approaches produced in Biomapper. In some cases, models improved by excluding particular ecogeographic variables that were not related to the species distribution (for example no species locations occurred on the ecogeographic variable). As such, some species were run iteratively, successively dropping ecogeographic variables that did not explain any variation in the species distribution. Mammal species used in our analysis are listed in S1 Table. Habitat suitability scores were summed for all species to produce the index of mammal biodiversity.

Birds. We used SDMs developed by Preston (unpublished) based on presence only data from a variety of sources for 6 bird species in southern California. We compiled location records from the United States Fish and Wildlife Service (CFWO 2014), the California Natural Diversity Database (CNDDB 2014), United States Geological Survey (USGS), Western Riverside County Multiple Species Habitat Conservation Plan Monitoring Program (WRC MSHCP 2014), San Diego County’s regional species database (SANBIOS 2014), Natural Community Conservation Plan surveys (NCCP; Rotenberrry unpub) and ORNIS online database ([www.ornisnet.org](http://www.ornisnet.org)). We constructed an environmental variable grid of points spaced 150 m apart across southern California and calculated climatic, topographic, and land cover variables at each grid point (TNC and SDMMP 2015, Preston and Kus 2015). We removed spatially redundant bird location records and used a spatially balanced subsampling strategy (Knick et al. 2013), randomly subsampling 20-50 locations by subregion in each of 1,000 iterations and averaging model results. We constructed alternative models with different combinations of environmental variables using the partitioned Mahalanobis D2 modeling approach (Rotenberry et al. 2002, 2006). We compared among habitat similarity index (HSI) median validation predictions ranging from 0 (least suitable) to 1.0 (most suitable) and AUC values to select the best performing model for each species. We then summed HSI values across species to obtain a predictor of species richness at each grid point.

**Genetic biodiversity**

To represent regional genetic biodiversity, we used previously developed raster models of intra-population sequence diversity and inter-population sequence divergence averaged across 14 and 21 small animal species, respectively (Vandergast et al. 2008, see S1 Table for included species). The genetic variation within species provides the raw material underlying a species’ ability to adapt to future environmental change. Genetic variation can be distributed both within and among populations, and patterns of diversity are often similar across species, shaped by regional biogeographic history (Avise 1992, Moritz and Faith 1998, Wood et al. 2013). Regions of high intrapopulation genetic diversity may be indicative of larger or more stable populations and provide evolutionary resilience (Soulé 1976, Frankham 1996, Mulligan et al. 2006) while regions of high genetic divergence (in the absence of geographic barriers) may be indicative of recontact zones between previously isolated lineages ([Barton et al. 1983](#_ENREF_3), [Nettel et al. 2008](#_ENREF_41)). Vandergast et al. (2008) interpolated continuous rasters from average sequence diversity measured within populations for 14 small animal species (S1 Table) for which mtDNA sequence data were collected throughout southern California. These were averaged into a single raster depicting regional hotspots of intrapopulation genetic diversity for the species assemblage. A similar approach was used to create a raster depicting regional hotspots of average genetic divergence for the species assemblage. Here, measured sequence divergence values between pairs of populations were mapped to the geographic midpoints between populations and interpolated into continuous rasters for 21 small animal species (S1 Table).

**S1 Table. Species included in biodiversity criteria.**

| Plants |
| --- |
| *Achillea millefolium, Acmispon glaber* var. *glaber, Acmispon strigosus, Acourtia microcephala, Adenostoma fasciculatum* var. *fasciculatum, Adenostoma sparsifolium, Adolphia californica, Ambrosia psilostachya, Amorpha fruticosa, Amsinckia menziesii, Anemopsis californica, Antirrhinum nuttallianum* subsp. *nuttallianum, Arctostaphylos glandulosa* subsp. *glandulosa, Arctostaphylos glauca, Arctostaphylos pungens, Artemisia californica, Artemisia douglasiana, Artemisia dracunculus, Artemisia tridentata* subsp. *tridentata, Atriplex canescens* var. *canescens, Baccharis salicifolia* subsp. *salicifolia, Baccharis sarothroides, Bebbia juncea* var. *aspera, Brickellia californica, Calystegia macrostegia* subsp. *macrostegia, Ceanothus crassifolius* var. *crassifolius, Ceanothus cuneatus* var. *cuneatus, Ceanothus greggii, Ceanothus leucodermis, Ceanothus oliganthus* var. *oliganthus, Ceanothus tomentosus, Cercocarpus betuloides* var. *betuloides, Chaenactis artemisiifolia, Chaenactis glabriuscula* var. *glabriuscula*, *Claytonia parviflora* subsp. *parviflora*, *Claytonia perfoliata* subsp. *perfoliata*, *Clematis lasiantha*, *Cneoridium dumosum*, *Crassula connata*, *Cressa truxillensis*, *Croton californicus*, *Cryptantha intermedia* var. *intermedia*, *Cryptantha muricata* var. *muricata*, *Daucus pusillus*, *Dendromecon rigida*, *Dichelostemma capitatum* subsp. *capitatum*, *Distichlis spicata*, *Dryopteris arguta*, *Dudleya edulis*, *Dudleya lanceolata*, *Dudleya pulverulenta*, *Elymus condensatus*, *Elymus glaucus* subsp. *glaucus*, *Emmenanthe penduliflora* var. *penduliflora*, *Encelia californica*, *Encelia farinosa*, *Eriodictyon crassifolium* var. *crassifolium*, *Eriogonum fasciculatum* var. *fasciculatum*, *Eschscholzia californica*, *Eucrypta chrysanthemifolia* var. *chrysanthemifolia*, *Frankenia salina*, *Galium angustifolium* subsp. *angustifolium*, *Galium aparine*, *Gutierrezia californica*, *Gutierrezia sarothrae*, *Hazardia squarrosa* var. *squarrosa*, *Helianthemum scoparium*, *Heliotropium curassavicum* var. *oculatum*, *Hesperocyparis forbesii*, *Heteromeles arbutifolia*, *Heterotheca grandiflora*, *Isocoma menziesii* var. *menziesii*, *Iva hayesiana*, *Keckiella antirrhinoides* var. *antirrhinoides*, *Keckiella cordifolia*, *Lasthenia californica* subsp. *californica*, *Lepidospartum squamatum*, *Logfia filaginoides*, *Lonicera subspicata* var. *subspicata*, *Lupinus bicolor*, *Malacothamnus fasciculatus* var. *fasciculatus*, *Malosma laurina*, *Melica imperfecta*, *Mimulus aurantiacus* var. *aurantiacus*, *Nemophila menziesii* var. *menziesii*, *Opuntia basilaris* var. *basilaris*, *Opuntia engelmannii* var. *engelmannii*, *Opuntia* *littoralis*, *Paeonia* *californica*, *Pentagramma* *triangularis* subsp. *triangularis*, *Peritoma* *arborea* var. *arborea*, *Phacelia* *distans*, *Phacelia* *ramosissima*, *Pinus* *contorta* subsp. *contorta*, *Plantago* *erecta*, *Platanus* *racemosa*, *Porophyllum* *gracile*, *Prunus* *ilicifolia* subsp. *ilicifolia*, *Pseudognaphalium* *biolettii*, *Pseudognaphalium* *californicum*, *Pterostegia* *drymarioides*, *Quercus* *agrifolia* var. *agrifolia*, *Quercus* *berberidifolia*, *Quercus* *dumosa*, *Quercus* *engelmannii*, *Quercus* *wislizeni* var. *wislizeni*, *Rhamnus* *crocea*, *Rhamnus* *ilicifolia*, *Rhus* *aromatica*, *Rhus* *ovata*, *Ribes* *indecorum*, *Ribes* *malvaceum* var. *malvaceum*, *Ribes* *speciosum*, *Rosa* *californica*, *Rubus* *ursinus*, *Salix* *exigua* var. *exigua*, *Salix* *gooddingii*, *Salix* *laevigata*, *Salix* *lasiolepis*, *Salvia* *apiana*, *Salvia* *columbariae*, *Salvia* *mellifera*, *Scrophularia* *californica*, *Selaginella* *bigelovii*, *Simmondsia* *chinensis*, *Sisyrinchium* *bellum*, *Solanum* *xanti*, *Stipa* *coronata*, *Stipa* *pulchra*, *Stylocline* *gnaphaloides*, *Toxicodendron* *diversilobum*, *Typha* *domingensis*, *Typha* *latifolia*, *Uropappus* *lindleyi*, *Vitis* *girdiana*, *Xanthium* *strumarium*, *Xylococcus* *bicolor*, *Yucca* *schidigera* |
| Herpetofauna |
| *Aspidoscelis hyperythra, Aspidoscelis tigris, Batrachoseps major, Batrachoseps nigriventris, Bufo boreas, Coleonyx variegatus, Coluber mormon, Crotalus oreganus, Crotalus ruber, Elgaria multicarinata, Eumeces gilberti, Eumeces skiltonianus, Hypsiglena torquata, Lampropeltis getula, Leptotyphlops humilis, Lichanura orcutti, Masticophis flagellum, Masticophis lateralis, Phrynosoma coronatum, Pituophis catenifer, Pseudacris regilla, Rhinocheilus lecontei, Salvadora hexalepis, Sceloporus occidentalis, Sceloporus orcutti, Spea hammondii, Tantilla planiceps, Thamnophis hammondii, Uta stansburiana* |
| Birds |
| *Artemisiospiza belli belli, Calypte costae, Campylorhynchus brunneicapillus, Chamaea fasciata, Polioptila californica californica, Toxostoma redivivum* |
| Mammals |
| *Canis latrans, Chaetodipus fallax, Dipodomys simulans, Lepus californicus, Lynx rufus, Mephitis mephitis, Microtus californicus, Neotoma lepida, Neotoma macrotis, Odocoileus hemionus, Peromyscus californicus, Peromyscus eremicus, Procyon lotor, Puma concolor, Reithrodontomys megalotis, Sylvilagus audubonii, Taxidea taxus, Urocyon cinereoargenteus* |
| Genetic Diversity and Divergence  *Bolded taxa included in both layers, non-bolded contributed to divergence layer only. |
| Invertebrates (4): ***Apomastus schlingeri/kristenae****,* ***Branchinecta sandiegonensis****,* ***Hemileuca electra****,* ***Stenopelmatus mahogani***  Amphibians (2): *Batrachoseps nigriventris, Batrachoseps major*  Reptiles (8): *Emys marmorata, Eumeces gilberti, Lampropeltis zonata,* ***Lichinura trivirgata****, Masticophis flagellum,* ***Phrynosoma coronatum, Sceloporus occidentalis****, Xantusia henshawi*  Birds (3): ***Chamaea fasciata****,* ***Picoides albolarvatus****,* ***Toxostoma redivivum***  Mammals (4): ***Neotoma fuscipes****,* ***Neotoma lepida****,* ***Perognathus longimembris****,* ***Sorex ornatus*** |

**References**

Avise, J. 1992. Molecular population structure and the biogeographic history of a regional fauna: A Case history with lessons for conservation biology. Oikos 63:62‒76.

Barton, N. H., R. B. Halliday, and G. M. Hewitt. 1983. Rare electrophoretic variants in a hybrid zone. Heredity 50:139‒146.

Cutler, D. R., T. C. Edwards, K. H. Beard, A. Cutler, K. T. Hess, J. Gibson, and J. J. Lawler. 2007. Random forests for classification in ecology. Ecology 88:2783‒2792.

Fisher, R. N., D. Stokes, C. J. Rochester, C. Brehme, S. A. Hathaway, and T. J. Case. 2008. Herpetological monitoring using a pitfall trapping design in southern California. U.S. Geological Survey, Western Ecological Research Center, Sacremento, California.

Frankham, R. 1996. Relationship of genetic variation to population size in wildlife. Conservation Biology 10:1500‒1508.

Franklin, J., K. E. Wejnert, S. A. Hathaway, C. J. Rochester, and R. N. Fisher. 2009. Effect of species rarity on the accuracy of species distribution models for reptiles and amphibians in southern California. Diversity and Distributions 15:167‒177.

Halsey, R. W. 2005. Fire, Chaparral, and Survival in Southern California. Sunbelt Publications, San Diego, California.

Hanley, J. A., and B. J. McNeil. 1982. The meaning and use of the area under a receiver operating characteristics curve. Radiology 143:29‒36.

Hijmans, R. J. 2013. raster: Geographic analysis and modeling with raster data.

Hirzel, A. H., J. Hausser, D. Chessel, and N. Perrin. 2002. Ecological-niche factor analysis: How to compute habitat-suitability maps without absence data? Ecology 83:2027‒2036.

Hunter, R. D., R. N. Fisher, and K. R. Crooks. 2003. Landscape-level connectivity in coastal southern California, USA, as assessed through carnivore habitat suitability. Natural Areas Journal 23:302‒314.

Knick, S. T., S. E. Hanser, and K. L. Preston. 2013. Modeling ecological minimum requirements for distribution of greater sage-grouse leks: implications for population connectivity across their western range, U.S.A. Ecology and Evolution 3:1539-1551.

Liu, C., P. M. Berry, T. P. Dawson, and R. G. Pearson. 2005. Selecting thresholds of occurrence in the prediction of species distributions. Ecography 28:385‒393.

Moritz, C., and D. P. Faith. 1998. Comparative phylogeography and the identification of genetically divergent areas for conservation. Molecular Ecology 7:419‒429.

Mulligan, C. J., A. Kitchen, and M. M. Miyamoto. 2006. Comment on ‘‘Population size does not influence mitochondrial genetic diversity in animals.” Science 314:1390a.

Nettel, A., R. S. Dood, Z. Afzal-Rafii, and C. Tovilla-Hernandez. 2008. Genetic diversity enhanced by ancient introgression and secondary contact in East Pacific black mangroves. Molecular Ecology 17:2680‒2690.

Phillips, S. J., M. Dudik, and R. E. Schapire. 2006. Maximum entropy modeling of species geographic distributions. Ecological Modelling 190:231‒259.

Preston, K. and B. Kus. 2015. Coastal California Gnatcatcher Proposed Regional Monitoring Sampling Design. Draft Report.

Principe, Z., J. B. MacKenzie, B. Cohen, J. M. Randall, W. Tippets, T. Smith, and S. A. Morrison. 2013. 50-Year Climate Scenarios and Plant Species Distribution Forecasts for Setting Conservation Priorities in Southwestern California. The Nature Conservancy of California, San Francisco, CA.

Rotenberry, J. T., S. T. Knick and J. E. Dunn. 2002. A minimalist approach to mapping species’ habitat: Pearson’s planes of closest fit. Pages 281-289 *in* J. M. Scott, P. J. Heglund, M. L. Morrison, J. B. Haufler, M. G. Raphael, W. A. Wall, and F. B. Samson, editors. Predicting Species Occurrences: Issues of Accuracy and Scale. Island Press, Washington, D.C., USA

Rotenberry, J. T., K. L. Preston, and S. T. Knick. 2006. GIS-based niche modeling for mapping species habitat. Ecology 87:1458‒1464.

Safford, H. D., K. van de Water, and D. Schmidt. 2011. California Fire Return Interval Departure (FRID) map, 2010 version. USDA Forest Service, Pacific Southwest Region and The Nature Conservancy-California.

Soulé, M. 1976. Allozyme variation: its determinants in space and time. Pages 60‒77 in F. J. Ayala, editor. Molecular Evolution. Sinauer, Sunderland, MA.

Syphard, A. D., and J. Franklin. 2010. Species’ traits affect the performance of species’ distribution models for plants in southern California. Journal of Vegetation Science 21:177‒189.

Syphard, A. D., and J. E. Keeley. 2015. Location, timing and extent of wildfire vary by cause of ignition. International Journal of Wildland Fire 24:37‒47.

The Nature Conservancy. 2013. Protecting Nature in San Diego County. The Nature Conservancy.

Vandergast, A. G., A. J. Bohonak, S. A. Hathaway, J. Boys, and R. N. Fisher. 2008. Are hotspots of evolutionary potential adequately protected in southern California? Biological Conservation 141:1648‒1664.

Wood, D. A., A. G. Vandergast, K. R. Barr, R. D. Inman, T. C. Esque, K. E. Nussear, and R. N. Fisher. 2013. Comparative phylogeography reveals deep lineages and regional evolutionary hotspots in the Mojave and Sonoran Deserts. Diversity and Distributions 19:722‒737.

Wood, S. 2006. Generalized Additive Models: An Introduction with R. CRC Press, Taylor and Francis Group, Boca Raton, FL.
