# Supplementary material for: Prioritizing conserved areas threatened by wildfire and fragmentation for monitoring and management
Source: PLoS One. 2018 Sep 7;13(9):e0200203. doi: 10.1371/journal.pone.0200203 (PMC6128460; doi:10.1371/journal.pone.0200203)
Supplement: S2 Appendix — (DOCX) [file pone.0200203.s003.docx]

**S2 Appendix: Illustrative Example of Pareto ranking.**

A simple example helps clarify Pareto ranking and the concept of weak Pareto dominance. In the table below, each grid cell, indexed by k, has three criteria on which they are ranked. Cell 1 has a rank of 1 because the values for all three criteria are less than the values in all other cells. Cells 2 and 3 do not dominate each other because they have equal values for criterion 2, but cell 2 has a lower value for criterion 1 while cell 3 has a lower value for criterion 3. Therefore, they receive the same rank. Cells 3 and 4 have the same rank because they are equal in all criteria. Cells 2 through 4, which all have a Pareto rank of 2, dominate cell 5 because their values for each of the criteria are less-than-or-equal-to the values for cell 5, but they have at least one value that is less. Thus, cell 5 receives the next rank of 3. Similarly, cell 5 dominates cell 6 because, although they are equal for criteria 1 and 3, cell 5 has a lower value for criteria 2. Thus, cell 6 receives the highest rank of 4.

**S2 Table. This table gives six vectors (indexed by k) of three criteria that are ranked against each other.** The Pareto rank is given in the last column.

| **Cell Index**  **(k)** | **Criteria** | | | Pareto  Rank |
| --- | --- | --- | --- | --- |
|  | **x_1,k_** | **x_2,k_** | **x_3,k_** |  |
| 1 | 0.1 | 0.1 | 0.1 | 1 |
| 2 | 0.2 | 0.2 | 0.3 | 2 |
| 3 | 0.3 | 0.2 | 0.2 | 2 |
| 4 | 0.3 | 0.2 | 0.2 | 2 |
| 5 | 0.3 | 0.2 | 0.4 | 3 |
| 6 | 0.3 | 0.3 | 0.4 | 4 |

Pareto Ranking Simulation Experiment

We conducted a simulation experiment to evaluate the effect of the number of criteria and the correlation among criteria on the Pareto ranking process. We varied the number of criteria from 3 to 9 and the covariance from -1.0 to 1.0. For each trial, we created a covariance matrix with 1.0 on the diagonal and set the off diagonal elements to the specified covariance for each experiment. If the covariance matrix was positive-definite, we randomly drew 500 vectors of criteria from a multivariate normal distribution and applied Pareto ranking to the result. We repeated this process 1000 times for each combination of number of criteria and covariance value and average the result. Below we show the results for the mean maximum Pareto rank across all 1000 trials (S1 Figure). This is an indicator of how well the ranking process resolves differences in the criteria sets. As we can see, the range of ranks assigned increases with (1) decreasing number of criteria in the set and (2) increasing covariance (and hence, correlation) among the criteria. Full R and C++ code is provided for the simulation experiment. The C++ function getParetoRanks(obj, verbose) can be used from within R to perform Pareto ranking and is the implementation we used in this paper.


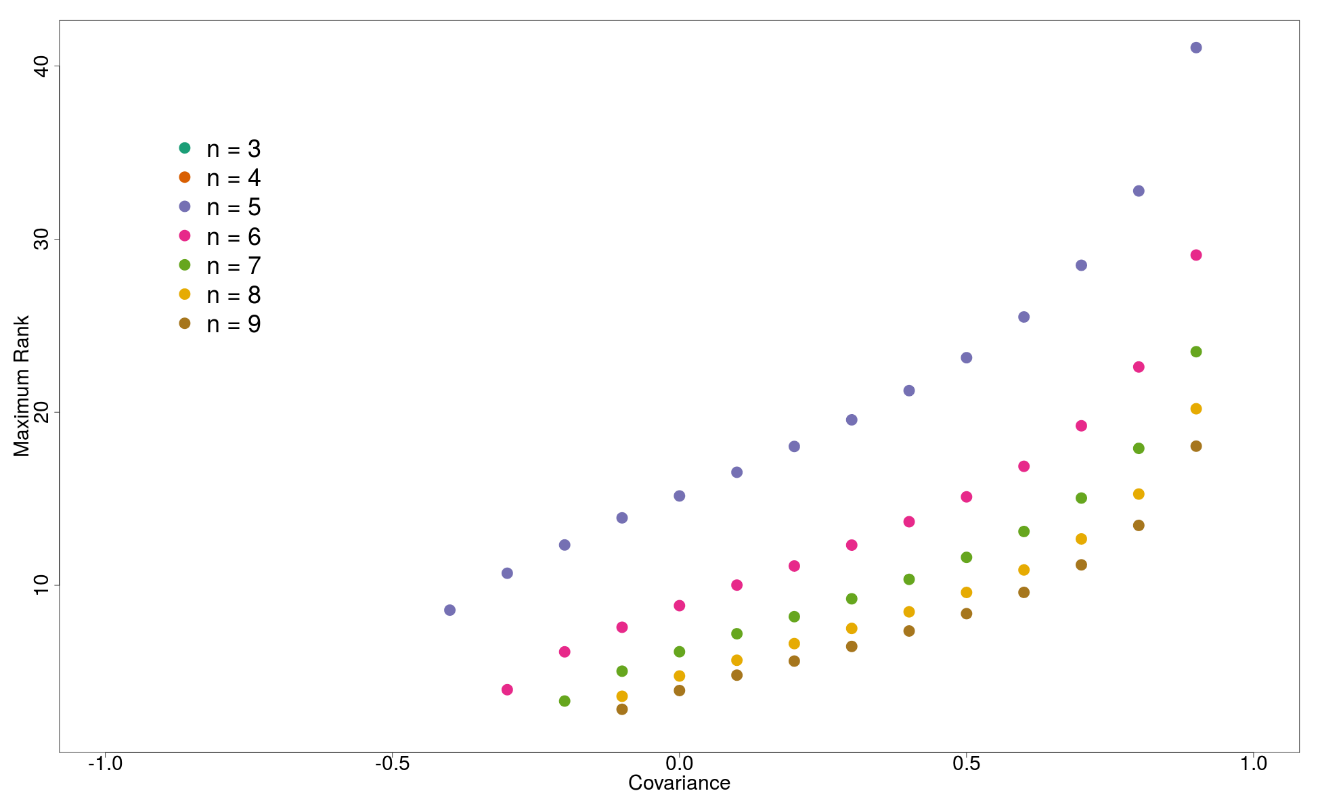


**S2 Figure: Mean maximum Pareto rank over 1000 trials for different combinations of number of criteria and covariance among criteria (given a positive-definite covariance matrix).** The colors correspond to number of criteria used in Pareto ranking as shown in the key.
