# Supplementary material for: Prioritizing conserved areas threatened by wildfire and fragmentation for monitoring and management
Source: PLoS One. 2018 Sep 7;13(9):e0200203. doi: 10.1371/journal.pone.0200203 (PMC6128460; doi:10.1371/journal.pone.0200203)
Supplement: S3 Appendix — (DOCX) [file pone.0200203.s004.docx]

**S3 Appendix: Summary Statistics and Correlation Coefficients for Criteria.**

**S3 Table A. A summary of threat criteria by climate zone and conservation status.** The mean is given, along with the standard deviation in parentheses, within each combination of climate zone and conservation status.

| **Climate Zone** | **Conserved** | **N** | **F***_Nfires_* | **F***_pr(ign)_* | **F***_FRID_* | **H***_road_* | **H***_dev_* | **H***_patch_* |
| --- | --- | --- | --- | --- | --- | --- | --- | --- |
| Maritime | Y | 15100 | 0.033 (0.179) | 0.061 (0.084) | 30.277 (22.363) | 0.194 (0.157) | 0.364 (0.275) | 272.425 (256.269) |
|  | N | 29874 | 0.141 (0.408) | 0.083 (0.122) | 17.361 (28.313) | 0.507 (0.228) | 0.771 (0.309) | 290.111 (386.871) |
|  | - | 44974 | 0.106 (0.354) | 0.081 (0.118) | 20.806 (27.455) | 0.468 (0.243) | 0.72 (0.333) | 283.264 (344.918) |
| Coastal | Y | 93230 | 1.377 (1.544) | 0.136 (0.169) | -20.523 (42.404) | 0.213 (0.166) | 0.238 (0.26) | 1962.885 (3877.29) |
|  | N | 372350 | 0.949 (1.283) | 0.15 (0.189) | -13.573 (39.522) | 0.309 (0.226) | 0.441 (0.379) | 3009.237 (4780.706) |
|  | - | 465580 | 1.032 (1.349) | 0.148 (0.186) | -15.416 (40.422) | 0.296 (0.221) | 0.413 (0.371) | 2786.268 (4623.192) |
| Transitional | Y | 296974 | 2.749 (1.432) | 0.21 (0.141) | -47.703 (25.948) | 0.067 (0.09) | 0.044 (0.117) | 5704.138 (6410.56) |
|  | N | 517768 | 1.809 (1.378) | 0.216 (0.181) | -30.503 (37.784) | 0.211 (0.178) | 0.278 (0.324) | 3311.371 (4263.752) |
|  | - | 814742 | 2.15 (1.469) | 0.214 (0.17) | -37.812 (34.343) | 0.168 (0.17) | 0.208 (0.298) | 4236.629 (5329.096) |
| Inland | Y | 702032 | 1.614 (1.084) | 0.177 (0.116) | -14.083 (38.446) | 0.035 (0.055) | 0.003 (0.018) | 23011.753 (31468.061) |
|  | N | 636761 | 1.313 (1.135) | 0.219 (0.163) | -0.945 (42.035) | 0.079 (0.085) | 0.024 (0.062) | 14478.217 (26351.868) |
|  | - | 1338793 | 1.471 (1.119) | 0.197 (0.142) | -8.281 (40.598) | 0.057 (0.074) | 0.013 (0.046) | 19072.234 (29525.714) |
| All | Y | 1107336 | 1.879 (1.344) | 0.181 (0.131) | -23.609 (38.786) | 0.063 (0.099) | 0.041 (0.132) | 16409.407 (26918.672) |
|  | N | 1556753 | 1.368 (1.301) | 0.194 (0.179) | -13.08 (42.308) | 0.217 (0.21) | 0.277 (0.355) | 7936.535 (18392.346) |
|  | - | 2664089 | 1.579 (1.343) | 0.189 (0.164) | -18.132 (40.995) | 0.164 (0.194) | 0.196 (0.318) | 11634.819 (22903.475) |

**S3 Table B. A summary of biodiversity criteria by climate zone and conservation status.** The mean is given, along with the standard deviation in parentheses, within each combination of climate zone and conservation status.

| **Climate Zone** | **Conserved** | **N** | **S***_plant_* | **S***_herp_* | **S***_bird_* | **S***_mamm_* | **G***_diverg_* | **G***_divers_* |
| --- | --- | --- | --- | --- | --- | --- | --- | --- |
| Maritime | Y | 15100 | 92.965 (10.918) | 8.173 (0.737) | 0.905 (0.685) | 2.5 (1.577) | 0.486 (0.025) | 0.092 (0.043) |
|  | N | 29874 | 86.889 (14.373) | 8.735 (0.905) | 1.045 (0.707) | 4.527 (2.102) | 0.489 (0.028) | 0.109 (0.073) |
|  | - | 44974 | 88.905 (13.637) | 8.552 (0.894) | 0.997 (0.703) | 3.809 (2.163) | 0.488 (0.027) | 0.107 (0.07) |
| Coastal | Y | 93230 | 104.24 (15.047) | 9.901 (1.3) | 2.507 (1.32) | 7.686 (1.677) | 0.517 (0.02) | 0.139 (0.054) |
|  | N | 372350 | 98.447 (14.948) | 9.93 (1.349) | 2.602 (1.236) | 8.25 (1.48) | 0.514 (0.017) | 0.175 (0.079) |
|  | - | 465580 | 99.607 (15.147) | 9.924 (1.339) | 2.583 (1.254) | 8.126 (1.543) | 0.515 (0.017) | 0.17 (0.077) |
| Transitional | Y | 296974 | 110.843 (16.496) | 9.9 (1.577) | 2.786 (1.173) | 8.849 (1.542) | 0.531 (0.024) | 0.171 (0.053) |
|  | N | 517768 | 104.845 (21.064) | 9.864 (1.814) | 2.96 (1.071) | 9.119 (1.222) | 0.54 (0.017) | 0.181 (0.053) |
|  | - | 814742 | 107.032 (19.735) | 9.876 (1.735) | 2.897 (1.112) | 9.007 (1.37) | 0.537 (0.02) | 0.178 (0.053) |
| Inland | Y | 702032 | 56.778 (24.603) | 7.444 (1.422) | 1.558 (0.987) | 4.597 (1.769) | 0.527 (0.052) | 0.251 (0.079) |
|  | N | 636761 | 58.64 (24.726) | 7.833 (1.458) | 1.67 (0.961) | 5.48 (2.026) | 0.538 (0.045) | 0.247 (0.085) |
|  | - | 1338793 | 57.666 (24.68) | 7.64 (1.453) | 1.611 (0.976) | 5.012 (1.945) | 0.532 (0.049) | 0.249 (0.082) |
| All | Y | 1107336 | 75.764 (33.293) | 8.371 (1.874) | 1.958 (1.211) | 5.945 (2.587) | 0.526 (0.044) | 0.217 (0.084) |
|  | N | 1556753 | 84.074 (30.211) | 9.053 (1.852) | 2.31 (1.222) | 7.175 (2.396) | 0.528 (0.033) | 0.196 (0.082) |
|  | - | 2664089 | 80.621 (31.793) | 8.786 (1.89) | 2.164 (1.23) | 6.626 (2.557) | 0.528 (0.037) | 0.203 (0.083) |

**S3 Table C. A summary of Pareto ranks by climate zone and conservation status.** The proportion of cells with ranks at or below the 25^th^ percentile is given, with the number of cells ranking in parentheses, within each combination of climate zone and conservation status. Matrices of fire, habitat fragmentation, species richness, and genetic biodiversity criteria are denoted as **F**, **H**, **S**, and **G**, respectively. The function r() refers to the ranks produced by Pareto ranking for a given input criteria matrix.

| **Climate Zone** | **Conserved** | **r(F\|S)** | **r(H\|S)** | **r(F\|G)** | **r(H\|S)** | **r(F\|S\|G)** | **r(H\|S\|G)** |
| --- | --- | --- | --- | --- | --- | --- | --- |
| Maritime | Y | 0.006 (1645) | 0.371  (6533) | 0.005  (1889) | 0.284  (9503) | 0  (1355) | 0.095  (6319) |
|  | N | 0.022  (967) | 0.411  (11417) | 0.016  (1539) | 0.556  (15490) | 0  (890) | 0.323  (11191) |
|  | - | 0.012  (2612) | 0.396  (17950) | 0.01  (3428) | 0.453  (24993) | 0  (2245) | 0.241  (17510) |
| Coastal | Y | 0.371  (55635) | 0.667  (72821) | 0.157  (61666) | 0.361  (84152) | 0.201  (55612) | 0.331  (72760) |
|  | N | 0.489  (70287) | 0.465  (252127) | 0.22  (86762) | 0.429  (308349) | 0.363  (70286) | 0.479 (252089) |
|  | - | 0.437  (125923) | 0.51  (324948) | 0.194  (148432) | 0.415  (392501) | 0.292  (125899) | 0.446  (324849) |
| Transitional | Y | 0.566  (231484) | 0.421  (241847) | 0.247  (267048) | 0.124  (279663) | 0.374  (231434) | 0.266  (241847) |
|  | N | 0.524  (301879) | 0.587  (336819) | 0.243  (359794) | 0.365  (448252) | 0.369  (301879) | 0.419  (336819) |
|  | - | 0.542  (533363) | 0.517  (578666) | 0.244  (626842) | 0.273  (727915) | 0.371  (533313) | 0.355  (578666) |
| Inland | Y | 0.05  (521778) | 0.007  (545459) | 0.218  (599705) | 0.111  (669119) | 0.191  (521641) | 0.074  (545364) |
|  | N | 0.099  (442185) | 0.046  (516517) | 0.32  (485647) | 0.277  (575106) | 0.303  (441746) | 0.204  (516235) |
|  | - | 0.073  (963964) | 0.026  (1061976) | 0.264  (1085361) | 0.188  (1244225) | 0.242  (963388) | 0.138  (1061599) |
| All | Y | 0.22  (810542) | 0.181  (866660) | 0.222  (930308) | 0.136  (1042437) | 0.244  (810042) | 0.15  (866290) |
|  | N | 0.29  (815318) | 0.308  (1116880) | 0.28  (933742) | 0.344  (1347197) | 0.332  (814801) | 0.332  (1116334) |
|  | - | 0.255  (1625862) | 0.252  (1983540) | 0.251  (1864063) | 0.254  (2389634) | 0.288  (1624845) | 0.253  (1982624) |

**S3 Table D. A summary of composite ranks by climate zone and conservation status.** The proportion of cells with ranks at or below the 25^th^ percentile is given, with the number of cells ranking in parentheses, within each combination of climate zone and conservation status. Matrices of fire, habitat fragmentation, species richness, and genetic biodiversity criteria are denoted as **F**, **H**, **S**, and **G**, respectively. The function r() refers to the ranks produced by Pareto ranking for a given input criteria matrix.

| **Climate**  **Zone** | **Conserved** | ***r*[*r*(F\|S) \| r(H\|S)]** | ***r*[*r*(F\|G) \| r(H\|G)]** | ***r*[*r*(F\|S\|G) \| r(H\|S\|G)]** |
| --- | --- | --- | --- | --- |
| Maritime | Y | 0.035  (1291) | 0.001  (1563) | 0  (1227) |
|  | N | 0.084  (812) | 0.002  (1243) | 0  (749) |
|  | - | 0.054  (2103) | 0.002  (2806) | 0  (1976) |
| Coastal | Y | 0.524  (51658) | 0.097  (56692) | 0.194  (51635) |
|  | N | 0.691  (60062) | 0.21  (72500) | 0.455  (60062) |
|  | - | 0.614  (111720) | 0.161  (129192) | 0.334  (111697) |
| Transitional | Y | 0.537  (221887) | 0.216  (255787) | 0.348  (221887) |
|  | N | 0.622  (274266) | 0.221  (323121) | 0.428  (274266) |
|  | - | 0.584  (496153) | 0.219  (578908) | 0.392  (496153) |
| Inland | Y | 0.013  (498219) | 0.229  (573546) | 0.118  (498219) |
|  | N | 0.059  (407475) | 0.348  (445444) | 0.25  (407372) |
|  | - | 0.034  (905694) | 0.281  (1018990) | 0.177  (905591) |
| All | Y | 0.198  (773055) | 0.216  (887588) | 0.189  (772968) |
|  | N | 0.318  (742615) | 0.287  (842308) | 0.332  (742449) |
|  | - | 0.257  (1515670) | 0.251  (1729896) | 0.259  (1515417) |

**S3 Table E. Matrix of correlation coefficients for cell criteria.**

|  | S_plant_ | S_bird_ | S_herp_ | S_mamm_ | G_diverg_ | G_divers_ | F_Nfires_ | F_pr(ign)_ | F_FRID_ | H_road_ | H_dev_ | H_patch_ |
| --- | --- | --- | --- | --- | --- | --- | --- | --- | --- | --- | --- | --- |
| S_plant_ |  | 0.604 | 0.7 | 0.813 | 0.015 | -0.442 | 0.324 | 0.112 | -0.436 | 0.17 | 0.229 | -0.277 |
| S_bird_ | 0.604 |  | 0.493 | 0.612 | 0.037 | -0.407 | 0.243 | 0.102 | -0.339 | 0.172 | 0.206 | -0.273 |
| S_herp_ | 0.7 | 0.493 |  | 0.697 | 0.134 | -0.287 | 0.204 | -0.012 | -0.353 | 0.142 | 0.206 | -0.14 |
| S_mamm_ | 0.813 | 0.612 | 0.697 |  | 0.017 | -0.377 | 0.321 | 0.098 | -0.394 | 0.235 | 0.245 | -0.313 |
| G_diverg_ | 0.015 | 0.037 | 0.134 | 0.017 |  | -0.425 | -0.013 | -0.148 | 0.009 | -0.037 | -0.004 | 0.038 |
| G_divers_ | -0.442 | -0.407 | -0.287 | -0.377 | -0.425 |  | -0.227 | -0.062 | 0.277 | -0.129 | -0.205 | 0.186 |
| F_Nfires_ | 0.324 | 0.243 | 0.204 | 0.321 | -0.013 | -0.227 |  | 0.194 | -0.758 | -0.161 | -0.16 | -0.129 |
| F_pr(ign)_ | 0.112 | 0.102 | -0.012 | 0.098 | -0.148 | -0.062 | 0.194 |  | -0.104 | 0.032 | -0.162 | -0.11 |
| F_FRID_ | -0.436 | -0.339 | -0.353 | -0.394 | 0.009 | 0.277 | -0.758 | -0.104 |  | 0.115 | 0.078 | 0.105 |
| H_road_ | 0.17 | 0.172 | 0.142 | 0.235 | -0.037 | -0.129 | -0.161 | 0.032 | 0.115 |  | 0.645 | -0.241 |
| H_dev_ | 0.229 | 0.206 | 0.206 | 0.245 | -0.004 | -0.205 | -0.16 | -0.162 | 0.078 | 0.645 |  | -0.145 |
| H_patch_ | -0.277 | -0.273 | -0.14 | -0.313 | 0.038 | 0.186 | -0.129 | -0.11 | 0.105 | -0.241 | -0.145 |  |
